# Symbiotic Variations among Wheat Genotypes and Detection of Quantitative Trait Loci for Molecular Interaction with Auxin-Producing *Azospirillum* PGPR

**DOI:** 10.3390/microorganisms11061615

**Published:** 2023-06-19

**Authors:** Jordan Valente, Florence Gerin, Agathe Mini, Rohan Richard, Jacques Le Gouis, Claire Prigent-Combaret, Yvan Moënne-Loccoz

**Affiliations:** 1 Univ Lyon, Université Claude Bernard Lyon 1, CNRS, INRAE, VetAgro Sup, UMR5557 Ecologie Microbienne, 43 Bd du 11 Novembre 1918, F-69622 Villeurbanne, France; jordan2512@gmail.com (J.V.); florence.gerin@univ-lyon1.fr (F.G.); claire.prigent-combaret@univ-lyon1.fr (C.P.-C.); 2 GDEC, INRAE, UCA, F-63000 Clermont-Ferrand, France; agathe.mini75@gmail.com (A.M.); rohan19937@gmail.com (R.R.); jacques.le-gouis@inrae.fr (J.L.G.)

**Keywords:** *Azospirillum*, GWAS, IAA, PGPR, QTL, root, wheat

## Abstract

Crop varieties differ in their ability to interact with Plant Growth-Promoting Rhizobacteria (PGPR), but the genetic basis for these differences is unknown. This issue was addressed with the PGPR *Azospirillum baldaniorum* Sp245, using 187 wheat accessions. We screened the accessions based on the seedling colonization by the PGPR and the expression of the phenylpyruvate decarboxylase gene *ppdC* (for synthesis of the auxin indole-3-acetic acid), using *gusA* fusions. Then, the effects of the PGPR on the selected accessions stimulating Sp245 (or not) were compared in soil under stress. Finally, a genome-wide association approach was implemented to identify the quantitative trait loci (QTL) associated with PGPR interaction. Overall, the ancient genotypes were more effective than the modern genotypes for *Azospirillum* root colonization and *ppdC* expression. In non-sterile soil, *A. baldaniorum* Sp245 improved wheat performance for three of the four PGPR-stimulating genotypes and none of the four non-PGPR-stimulating genotypes. The genome-wide association did not identify any region for root colonization but revealed 22 regions spread on 11 wheat chromosomes for *ppdC* expression and/or *ppdC* induction rate. This is the first QTL study focusing on molecular interaction with PGPR bacteria. The molecular markers identified provide the possibility to improve the capacity of modern wheat genotypes to interact with Sp245, as well as, potentially, other *Azospirillum* strains.

## 1. Introduction

Symbiotic interactions with Plant Growth-Promoting Rhizobacteria (PGPR) are important for plant growth and health [1,2]. These PGPR, especially from the genera *Azospirillum*, *Pseudomonas*, *Herbaspirillum* or *Bacillus*, benefit plants via biological nitrogen fixation, phosphorus solubilization, the production of phytohormones or antimicrobial compounds and/or by eliciting systemic resistance pathways [3,4,5,6,7].

Plant response to PGPR inoculation can vary depending on the environmental/agronomic conditions, PGPR features and plant host genotype [8,9,10,11]. For the latter, differences may be expected between plant species and between plant varieties within species in relation to particular root system architectures, root surface properties and rhizodeposition (root exudation) patterns, which can impact root colonization by PGPR and their gene expression patterns [12,13,14,15,16]. Thus, many studies have shown the effect of the plant genotype on the bacterial colonization of roots and the composition of the rhizosphere bacterial community, including for bacterial taxa known to include PGPR strains [17,18,19,20,21,22]. A few studies have also evidenced differential bacterial gene expression according to the plant host genotype, notably for genes such as *acdS* (1-aminocyclopropane-1-carboxylate deaminase) or *nifH* (nitrogen fixation), which are involved in plant-beneficial functions [10,20,23,24].

In the case of crops, key events determining the properties of the current varieties include domestication [25,26,27,28], and in the second half of the last century, the advent of modern breeding, which typically aims at developing high-yield cultivars able to value farming inputs, i.e., under conditions close to the agronomic optimum [29,30,31]. Modern cultivars may differ from ancient plant genotypes in terms of their root exudation because of differences in the physiology and metabolic composition of their plant tissues [32,33,34], root architecture [32,35,36] and disease resistance [37,38,39], which might lead to particular rhizobacterial community composition [17,21,40,41,42,43]. Indeed, interaction analysis of 192 bread wheat accessions with the 2,4-diacetylphloroglucinol (DAPG)-producing PGPR *Pseudomonas ogarae* F113 showed that the abilities for PGPR root colonization and expression of the DAPG genes *phl* on roots were higher overall in ancient wheat genotypes than modern genotypes, even though the capacity for PGPR interaction was maintained in certain modern cultivars [44]. However, *P. ogarae* F113 is a particular type of PGPR in terms of its taxonomy (γ-Proteobacteria), the bioactive metabolites produced (siderophore, hydrogen cyanide and DAPG) and its key enzymatic functions (1-aminocyclopropane-1-carboxylate deaminase), as well as its resulting plant-beneficial effects (phytoprotection from pathogens and plant-growth promotion) [10,45,46]. Whether other types of PGPR would have the same ability as strain F113 to interact with different wheat genotypes is not documented. It is an important issue in terms of understanding rhizosphere ecology, as well as for its practical implications for breeding strategies aiming to make use of symbiotic microbial partners, as plant roots are exposed to a diversified range of PGPR taxa in soil. This is particularly relevant when considering auxin-producing PGPR bacteria as they represent an important group of PGPR [4,15]. In this work, we address this issue with the α-proteobacterium *Azospirillum baldaniorum* (previously *brasilense*) Sp245, a model PGPR strain in which the bacterial synthesis of indole-3-acetic acid (IAA) has been extensively studied [15,47,48,49,50,51,52,53].

The analysis of crop–PGPR interactions is usually carried out with a very limited range of plant genotypes, but in recent years, larger-scale investigations have also been performed, which opens the door to implement genome-wide association studies (GWAS) and identify relevant Quantitative Trait Loci (QTL). GWAS can be useful to decipher the assembly of the rhizosphere microbiota, e.g., in maize [54] and foxtail millet [55]. QTL analyses have also been performed to detect the genomic regions involved in functional traits (i) to which microorganisms contribute, e.g., nitrogen use efficiency [56], or (ii) corresponding to key plant-microbe interactions, such as *Fusarium* head blight resistance [57] and arbuscular mycorrhizal symbiosis in *Triticum aestivum* [58,59]; arbuscular mycorrhizal symbiosis in *Triticum durum* [60]; growth response of *Arabidopsis thaliana* to *Pseudomonas simiae* WCS417r [61] or *Azoarcus olearius* DQS-4T [62]; and the effects of *Azospirillum brasilense* Ab-V5 on *Zea mays* [63]. However, no investigation has been performed to date that targets the molecular interactions of PGPR bacteria with roots, and this was a key aspect of this work.

The objective of the present study was to assess whether the IAA-producing PGPR *A. baldaniorum* Sp245 interacted differently with modern vs. ancient wheat genotypes, and to implement a GWAS to explore the genomic fragments potentially implicated in wheat– Sp245 interactions. To this end, a collection of 187 accessions of bread wheat and a gnotobiotic screening protocol derived from Valente et al. [44] were used to quantify root colonization by *A. baldaniorum* Sp245 and the expression of Sp245′s phenylpyruvate decarboxylase gene *ppdC* (for IAA synthesis [15,48]) on roots, based on fluorimetric monitoring of the reporter gene *gusA*. A greenhouse experiment was then conducted using a selection of eight wheat genotypes identified as either stimulating Sp245 or not in the gnotobiotic screening to compare their ability to benefit from Sp245 inoculation in non-sterile soil under an optimum or stress condition. Finally, a GWAS was performed to identify the wheat chromosome regions shared by accessions interacting well with *A. baldaniorum* Sp245.

## 2. Materials and Methods

### 2.1. Plant Material and Bacterial Strains

This study was carried out using the collection of 199 bread wheat (*Triticum aestivum*) accessions described in Valente et al. [44], which includes 35 landraces, 43 old varieties (≤1960) and 121 modern genotypes (>1960) developed after the introduction of *Rht-B1b (Rht1)* and *Rht-D1b (Rht2)* alleles from the Japanese variety Norin 10, conferring a lower gibberellin response [64]. However, 12 genotypes showed poor germination and the analysis was conducted with 33 landraces, 40 old varieties (≤1960) and 114 modern genotypes (>1960), i.e., 187 accessions (Table S1).

For the in vitro screening experiment, two plasmidic derivatives of *A. baldaniorum* Sp245 (kindly provided by the Centre of Microbial and Plant Genetics, University of Leuven, Belgium) were used. The first derivative, Sp245(pFAJ31.2), contains a plasmidic fusion between an un-characterized constitutive promoter and *gusA*, and the second derivative, Sp245(pFAJ64), is a fusion between the *ppdC* promoter and *gusA* [12,47]. To prepare the inoculum, the two derivatives were grown separately in nitrogen-free broth [65] supplemented with LBm (final concentration 2.5%) and tetracycline (final concentration 10 µg/mL) for 24 h at 27 °C, with shaking at 180 rpm. 

For the greenhouse pot experiment, the wild-type *A. baldaniorum* Sp245 was used and grown the same way as described above (but without adding tetracycline). 

### 2.2. Inoculation and Plant Growth Conditions in the Screening Experiment

Wheats seeds were surface-disinfected through successive immersions in 70% ethanol for 1 min, then in sodium hypochlorite solution (Na_2_CO_3_ 0.1 g, NaCl 3 g and NaOH 0.15 g in 100 mL distilled water supplemented with 10% commercial bleach containing 9.6% of active chlorine and 0.01% of Tween 20) for 40 min at 180 rpm, followed by rinsing three times (5 min each, with strong manual agitation) in sterile water and a final last bath in sterile water for 60 min (adapted from Pothier et al. [66]). Seeds were then transferred on plates containing sterile agar for plant culture (Agar A7921; Sigma-Aldrich, Saint-Quentin Fallavier, France) at 8 g/L, which were incubated for 24 h in the dark at 27 °C. The one-day-old seedlings were each placed in a sterile 50 mL glass tube (Ø 19.3 × 200 mm) containing 20 mL of sterile semi-solid agar for plant culture at 2 g/L. Then, three seeds of each genotype were inoculated with 100 µL of cell suspension of Sp245(pFAJ31.2), and three others with 100 µL of cell suspension of Sp245(pFAJ64). Each 100 µL bacterial suspension contained 4 × 10^7^ cells previously washed in sterile 10 mM MgSO_4_ solution. Controls received 100 µL of 10 mM MgSO_4_ solution per seed. The tubes were placed in a growth chamber for seven days at 21 °C, with a 16/8 h day/night cycle and 60% hygrometry.

### 2.3. Root Sampling and GUS Assays

For the screening experiment, 4-MethylUmbelliferyl-β-D-Glucuronide (MUG) was used as a substrate for GUS activity, which can be quantified through spectrofluorimetry of the reaction product, 4-methylumbelliferone (4-MU) [67]. The GUS activity from Sp245(pFAJ31.2) was measured to estimate the Sp245 colonization level on roots, while the GUS activity from Sp245(pFAJ64) was used to evaluate *ppdC* expression in Sp245 cells. At seven days of growth, plants were removed from the tubes and each root system was cut off in 1 cm fragments and put in a 2 mL Eppendorf tube. Then, 1.5 mL of an extraction solution for GUS assay (i.e., phosphate buffer 50 mM at pH 7, EDTA 10 mM, Sarkosyl 0.1%, Triton X-100 0.1%, 2-mercaptoethanol 1 mM) supplemented with MUG at 0.35 mg/mL was added to each tube. The tubes were strongly shaken for 5 min using a Vortex and put for 4 h in the dark at 37 °C. After the incubation, 200 µL of supernatant from each tube was transferred into the wells of black 96-well plates with clear bottoms. The GUS activity was measured using an Infinite M200 pro microplate reader (Tecan, Männedorf, Switzerland), with an excitation at 365 nm and an emission at 455 nm, to quantify the fluorescence intensity from 4-methylumbelliferone (cleaved MUG). For each wheat genotype, the mean fluorescence intensity of non-inoculated plants was subtracted from the data of inoculated plants. Due to substantial intra-genotype variability, the median data were used rather than the means.

### 2.4. Greenhouse Soil Experiment

Eight genotypes differing in their ability to interact with *A. baldaniorum* Sp245 in vitro were selected from the 187 accessions to compare the plant growth promotion effects of *A. baldaniorum* Sp245 in non-sterile soil under greenhouse pot conditions. They included two modern genotypes (Amifort and D130-63) and two old varieties (Concurrent and Coronation) with effective interactions in vitro with Sp245, as well as two modern genotypes (Danubia and Hendrix) and two old varieties (Jaszaji TF and Odesskaya 16) not effective in their interactions with Sp245.

Wheat seeds were surface-disinfected in sodium hypochlorite, as described above (but without using ethanol). They were put in pots containing 1700 g of sieved non-sterile soil taken from the topsoil (loamy texture, organic matter 5.5%, pH 6.0) of a luvisol at La Côte Saint-André (France), with a soil water content of 22% *w*/*w*. Each seed was then inoculated with 200 µL of cell suspension of *A. baldaniorum* Sp245 containing 2 × 10^7^ cells or received 200 µL of MgSO_4_ 10 mM (in the controls). At day 14, a combined stress was applied to half the plants by stopping watering until the water content was 12% *w*/*w* and maintaining this level until the end of the experiment without the addition of any nutrient solution. For the optimum conditions, the soil water content was maintained at 22% *w*/*w* and a NPK nutrient solution (Plant-Prod 20-20-20; Plantproducts, Leamington, ON, Canada) was used to bring 8 mg N per plant on day 14 and on day 21. Eight plants were grown per genotype for each of the four inoculated/control × stress/optimum treatment combinations (randomized block design, with two plants per pot), and the experiment was carried out in a greenhouse with a 16/8 h day/night cycle at, respectively, 24 °C/20 °C and 40%/60% hygrometry.

On day 28, plants were removed from the soil and each root system was carefully cut off and washed. Each of the eight plants per treatment was used to determine the biomass of the roots and shoots, and then to characterize the root architecture using WinRhizo image analysis software (Regent Instruments, Nepean, ON, Canada); finally, the plant parts were dried 48 h at 105 °C and weighed again.

### 2.5. Genotyping Data

The accessions were genotyped with an Affymetrix SNP array [68]. Among the 423,385 single-nucleotide polymorphism (SNP) markers, 31,340 are labelled as Off-Target Variant (OTV) markers. Along the SNP alleles and eventually missing data (NA), they show a null allele (OTV) with no amplification. As suggested by Didion et al. [69], they can be used to detect presence/absence variations (PAV). In order to take them into account, the OTV markers were split into two markers: a SNP (OTV were transformed into NA) and an OTV (encoded with A for the presence of the fragment, and T for the absence of the fragment). Before data cleaning, there were 454,725 markers in the matrix. 

Heterozygous genotypes were replaced by NA. Markers that were monomorphic (114,562 markers) or with a NA percentage superior to 10% (30,124 markers) were deleted from the matrix. Imputation of NA was then performed with the Beagle v4.1 software [70] using standard parameters. SNP without a physical position (16,985 markers) on Chinese Spring RefSeqv1.0 [71] were first removed because the Beagle software cannot impute unmapped markers. After imputation, markers with a Minor Allele Frequency (MAF) inferior to 5% (53,358 markers) were deleted from the matrix. In the end, there were 239,696 SNP, among which 230,325 are public and have been used for GWAS.

### 2.6. Genome Wide Association Study

GWAS was performed with R package GenABEL [72], and more specifically, with the “polygenic” [73] and the “mmscore” functions [74]. The model used for GWAS was:

Y = µ + Xβ + G + E

where Y is the vector of the phenotypic values, µ is the overall mean, X the vector of SNP scores, β the additive effect of the SNP, and G and E are the random polygenic and residual effects. As proposed by Rincent et al. [75], the random polygenic effect was modelled with a genomic kinship matrix derived from all the SNP, except those on the chromosome being tested. In order (i) to remove redundant SNP (and consequently, shorten calculation time) and (ii) not to allocate too much weight to SNP in LD, particularly SNP located around centromeres, the matrix was pruned before computing. For each chromosome, the Linkage Disequilibrium (LD) of each pair of SNP within 600 kb was estimated, and if the r^2^ was superior to 0.9, one SNP of the pair was removed. The kinship matrix was then calculated as proposed by VanRaden [76].

To control for the effect of multiple testing, the number of independent tests was calculated as in Li and Ji [77] and a −log_10_(*P*) of 5 was then considered to identify significant marker-trait associations. To define the number of regions by chromosome, significant markers were clustered by the average distance on LD using a cut-off at (1-“critical LD”). The critical LD was estimated as the 99.9th percentile of the LD distribution assessed on 21,000 randomly chosen pairs of unlinked loci (mapped on different chromosomes). A Khi^2^ test was used to assess the hypothesis that three accessions classes (landraces, ≤1960, >1960) had the same marker alleles proportions.

### 2.7. Statistics

Absolute deviations (AD) and standard errors (SE) were used to display data fluctuations when using the median and mean data, respectively. Comparisons between the screening data were performed using Kruskal-Wallis rank-sum tests, followed by Conover-Iman tests for pairwise comparisons, as the data could not been normalized. When only two treatments were conducted, comparisons were carried out using Wilcoxon rank-sum tests. Comparisons of multiple and pairwise proportions were conducted using Khi^2^ tests and Z-tests, respectively. The plant data were normalized using optimizing box-cox transformation and compared using multiple-factors ANOVA, followed by Fisher’s LSD tests. The analyses were conducted using the Xlstat software v2018.4 (Addinsoft, Bordeaux, France) at *p* < 0.05 level. 

## 3. Results

### 3.1. Sp245 Colonization of Wheat in the Screening Experiment

**Figure 1:**
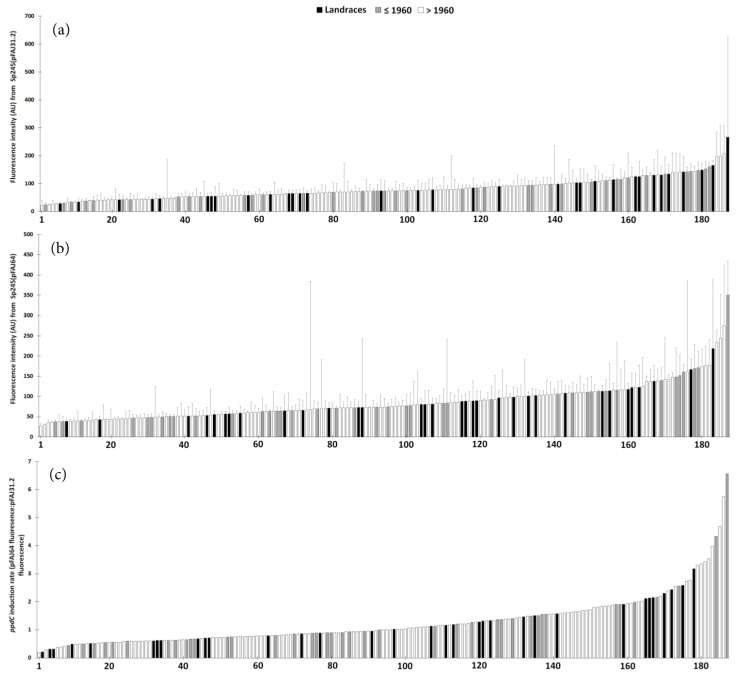
Colonization of *A. baldaniorum* Sp245(pFAJ31.2) and *ppdC* expression of Sp245(pFAJ64) on the roots of 187 individual wheat genotypes corresponding to 114 modern (>1960) and 73 ancient wheat genotypes (including 33 landraces and 40 old varieties [≤1960]). Fluorescence from 4-MU is shown in (**a**) for the pFAJ31.2 plasmid (root colonization) and in (**b**) for the pFAJ64 plasmid (*ppdC* expression), whereas the *ppdC* induction rate (pFAJ64 fluorescence:pFAJ31.2 fluorescence ratio) is given in (**c**). Fluorescence is expressed as arbitrary units (AU) and data are presented as means (n = 3) with standard errors. The corresponding ranking of the 187 genotypes is detailed in Table S1.

**Figure 2:**
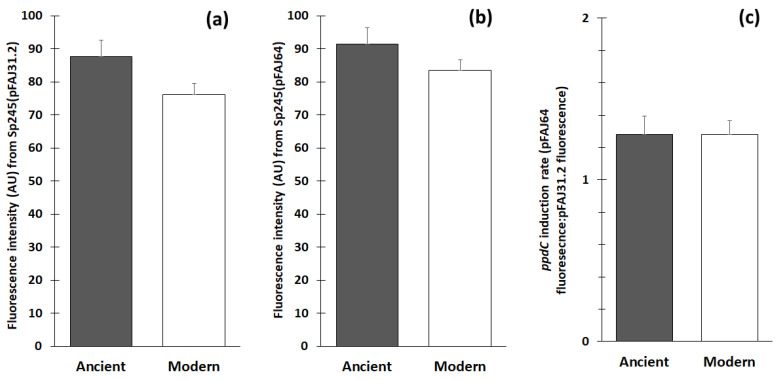
Colonization of *A. baldaniorum* Sp245(pFAJ31.2) and *ppdC* expression of Sp245(pFAJ64) on the roots of modern (n = 114) and ancient wheat genotypes (n = 73). Fluorescence from 4-MU is shown in (**a**) for the pFAJ31.2 plasmid (root colonization) and in (**b**) for the pFAJ64 plasmid (*ppdC* expression), whereas the *ppdC* induction rate (pFAJ64 fluorescence:pFAJ31.2 fluorescence ratio) is given in (**c**). Fluorescence is expressed as arbitrary units (AU) and data are presented as means (computed from individual genotype data) with standard errors. There was no significant difference at *p* < 0.05.

Root colonization by *A. baldaniorum* under simplified conditions was assessed using the derivative Sp245(pFAJ31.2), which contains a plasmidic fusion between an un-characterized constitutive promoter and the reporter gene *gusA* [12]. The analysis of the GUS activity related to root colonization by *A. baldaniorum* Sp245(pFAJ31.2) gave median fluorescence intensities that ranged between 22 ± [AD] 19 and 266 ± 358 arbitrary units (AU), depending on the wheat genotype (Figure 1). The data for the modern genotypes, old varieties and landraces were 76 ± [SE] 3, 84 ± 6 and 92 ± 8 AU (Figure S1), respectively, but the significance level was only *p* = 0.07 when comparing the 114 modern genotypes with all 73 ancient genotypes (i.e., including old varieties and landraces) (Figure 2). However, 20.5% and 38.4% of the ancient genotypes were among the 25 and 50 most colonized genotypes, respectively, versus only 8.7% and 19.3% of the modern genotypes (both at *p* < 0.05) (Table 1 and Table S2).

### 3.2. ppdC Expression on Wheat in the Screening Experiment

The expression of the IAA gene *ppdC* by root-colonizing *A. baldaniorum* was investigated using the derivative Sp245(pFAJ64), which contains a plasmidic fusion between the *ppdC* promoter and *gusA* [47]. For the *ppdC* expression analysis, the median fluorescence intensities varied between 27 ± [AD] 5 and 351 ± 84 AU between genotypes (Figure 1b). The differences were not significant when comparing the 114 modern (83 ± [SE] 4 AU) vs. the 73 ancient genotypes (91 ± 6 AU) (Figure 2). There was a significant difference when comparing the prevalence of ancient vs. modern genotypes among the 25 least colonized genotypes (respectively, 6.8% vs. 17.4%, *p* < 0.05), but not among the most colonized ones (Table 1 and Table S2). The correlation between Sp245 colonization and *ppdC* expression data (n = 190) was significant (*p* < 0.05) but weak (Spearman’s r = 0.19). 

The *ppdC* induction rate (i.e., the median fluorescence intensity in the Sp245(pFAJ64) treatment divided by the median fluorescence intensity in the Sp245(pFAJ31.2) treatment) ranged between 0.19 and 6.57 depending on the genotype (Figure 1). There was no difference in the *ppdC* induction rate between the 114 modern (1.28 ± 0.08) and 73 ancient genotypes (1.28 ± 0.11) (Table 1).

### 3.3. Impact of Stress on Wheat Genotypes of Different Interaction Abilities

**Figure 3:**
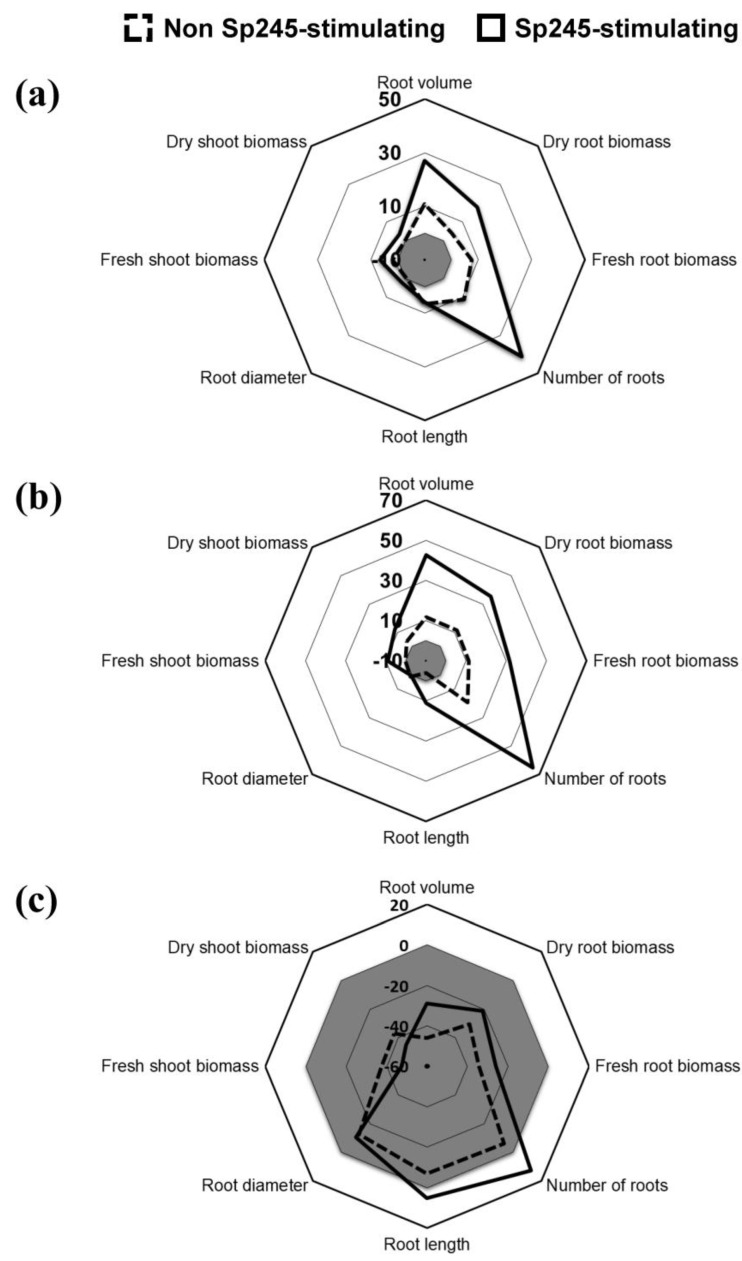
Relative impact of seed inoculation with *A. baldaniorum* Sp245 on the growth of four non-Sp245-stimulating and four Sp245-stimulating wheat genotypes under optimum (**a**) or combined stress conditions (**b**), and relative impact of stress on performance of non-inoculated plants (**c**). The relative impacts were computed as (inoculated − non-inoculated)/non-inoculated [in **a**,**b**], and (stress − optimum)/optimum [in **c**] for each of the eight plant parameters investigated. The grey areas correspond to negative values.

A genotype × stress two-factor ANOVA performed on the dataset from non-inoculated plants showed that stress had a significant impact (*p* < 0.001) on every plant parameter (except the number of roots), with a significant interaction between genotype and stress for the root average diameter and dry root biomass (*p* < 0.05). Depending on the genotype, the effect of stress was significant on two to five of the eight plant parameters studied (Table S3). Overall, the impact of stress on the four genotypes effective at interacting with Sp245 (hereafter referred to as Sp245-stimulating genotypes) vs. the four genotypes ineffective at interacting with Sp245 (hereafter referred to as non-Sp245-stimulating genotypes) was −29.0 ± [SE] 4.2% vs. −46.0 ± 8.1% for root volume, −10.4 ± 6.5% vs. −12.6 ± 5.4% for root average diameter, −26.1 ± 3.5% vs. −34.4 ± 9.3% for fresh root biomass, −21.3 ± 1.7% vs. −30.3 ± 9.8% for dry root biomass, −47.4 ± 3.8% vs. −37.5 ± 6.1% for fresh shoot biomass and −45.2 ± 5.4% vs. −36.9 ± 6.8% for dry shoot biomass (Figure 3); however, none of these differences was significant at *p* < 0.05 level.

### 3.4. Stimulation Effects of A. baldaniorum Sp245 on Wheat Genotypes of Different Interaction Abilities

The results of the three-factor (genotype × stress × inoculation) ANOVA showed that Sp245 inoculation had a significant effect on the root volume (*p* < 0.01), the number of roots (*p* < 0.001) and the fresh (*p* < 0.001) and dry root biomass (*p* < 0.001).

Under optimum condition, inoculation led to a significant increase in the number of roots (+94.6%) and in dry root biomass (+38.9%) of the Sp245-stimulating genotype Concurrent, whereas the positive effects on other genotypes were not significant (Table S4). Overall, the plant parameters in the inoculated treatments for the Sp245-stimulating vs. non-Sp245-stimulating genotypes were +26.9 ± [SE] 14.2% vs. +10.7 ± 3.5% for the root volume, +41.3 ± 20.3% vs. +11.0 ± 4.1% for the number of roots, +15.6 ± 6.2% vs. +7.5 ± 3.7% for fresh root biomass and +17.8 ± 7.4% vs. +4.2 ± 5.6% for dry root biomass in comparison with the controls (Figure 3).

Under stress, Sp245 inoculation had significant positive effects on three of the four Sp245-stimulating genotypes when considering the root volume of Concurrent (+75.8%) and Amifort (+49.6%), the number of roots of Concurrent (+80.6%), Amifort (+86.3%) and Coronation (+88.3%), fresh root biomass of Concurrent (+54.2%) and Amifort (+43.2%) and dry root biomass of Concurrent (+57.0%) and Amifort (+52.2%), but with no significant effect on any of the four non-Sp245-stimulating genotypes (Table S4). Thus, in comparison with the non-inoculated controls, the growth parameters upon inoculation of the Sp245-stimulating vs. non-Sp245-stimulating genotypes were +42.8 ± (SE) 14.2% vs. +11.8 ± 2.7% for the root volume, +65.4 ± 19.7% vs. +19.4 ± 6.3% for the number of roots, +31.7 ± 10.3% vs. +11.3 ± 3.1% for fresh root biomass and +35.5 ± 11.8% vs. +11.6 ± 3.4% for dry root biomass (Figure 3).

### 3.5. Wheat Stimulation by A. baldaniorum Sp245 vs. Pseudomonas ogarae F113

When the data in Valente et al. [44] on the interaction of the same eight wheat genotypes with *P. ogarae* F113 were included, the correlation between the mCherry red fluorescence intensity (F113 colonization) and 4-MU fluorescence intensity (Sp245 colonization) was not significant, regardless of whether ancient (n = 73), modern (n = 114) or all 187 genotypes were considered. Among the 50 genotypes most colonized by *P. ogarae* F113 and the 50 most colonized by *A. baldaniorum* Sp245 (making 86 genotypes in total, of which 23 landraces, 21 old varieties and 42 modern genotypes), only 14 of them (i.e., 16.3%) were common to both lists. These 14 genotypes included 3 of the 23 landraces (13.0%), 7 of the 21 old varieties (33.3%) and 4 of the 42 modern genotypes (9.5%) among the 86 most colonized genotypes, and only the difference between the old varieties and modern genotypes was significant (*p* < 0.05) (Table 2). In contrast, for the 50 least colonized genotypes by Sp245 and/or F113 (85 in total), the 15 genotypes common to the Sp245 and F113 lists included 0 of the 14 landraces, 2 of the 17 old varieties (11.8%) and 13 of the 54 modern genotypes (24.1%), and only the difference between the landraces and modern genotypes was significant (*p* < 0.05) (Table 2).

The correlation between EGFP green fluorescence (*phl* expression) and 4-methylumbelliferone fluorescence intensity (*ppdC* expression) was not significant when assessing 73 ancient, 114 modern or all 187 genotypes. Among the 50 genotypes showing the highest *phl* expression and the 50 with the highest *ppdC* expression (making 88 genotypes in total, of which 18 landraces, 24 old varieties and 46 modern genotypes), only 12 of them (i.e., 13.6%) were present in both lists. These 12 genotypes included 2 of the 18 landraces (11.1%), 7 of the 24 old varieties (29.2%) and 3 of the 46 modern genotypes (6.5%), and only the difference between the old varieties and modern genotypes was significant (*p* < 0.05). Among the 50 genotypes showing the lowest expression of *ppdC* or *phl* (87 in total), there were 13 genotypes common to the *ppdC* and *phl* lists, which included 1 of the 14 landraces (7.1%), 6 of the 16 old varieties (37.5%) and 6 of the 57 modern genotypes (10.5%). The differences between the old varieties and (i) modern genotypes or (ii) landraces were significant (*p* < 0.05) (Table 2).

### 3.6. Genome-Wide Association Study with A. baldaniorum Sp245 Data

**Figure 4:**
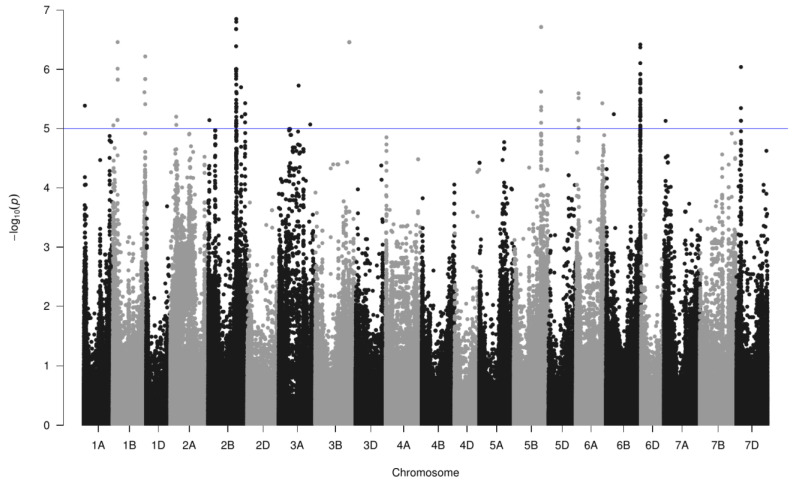
Manhattan plot for GWAS conducted on 180 bread wheat accessions for *A. baldaniorum* Sp245 root colonization, *ppdC* expression and *ppdC* induction rate (considering the means and the medians) with 230,325 SNP markers. The −log_10_(*P*) values are plotted against their respective physical positions on each chromosome of the Chinese Spring RefSeqv1.0. The blue line is used to highlight the −log_10_(*P*) threshold of 5.

A GWAS was conducted on root colonization by *A. baldaniorum* Sp245, *ppdC* expression and *ppdC* induction rate (considering the means and the medians) with 230,325 SNP (Figure 4). Using a −log_10_(*P*) superior to 5 led to the identification of 169 significant marker-trait associations distributed on 11 chromosomes (Table 3). No significant association was observed for root colonization. After clustering the 157 markers involved using a critical LD of 0.168, we identified 12 regions for *ppdC* expression in Sp245, seven for *ppdC* induction rate and three common to the two traits. In all cases, the minor allele increased the value of the trait (Table S5). The SNP with the largest effect is in group 19 on chromosome 6B for *ppdC* expression (+49 AU for the minor allele) and in group 15 on chromosome 3B for the *ppdC* induction rate (+1.12 for the minor allele). Nine markers showed a highly significant (*p* < 0.01) difference for the allele proportion between the three accession classes (landraces, ≤1960, >1960). This concerned four of the five markers in group 5 on chromosome 1B, the marker in group 7 on chromosome 2B, three of the 60 markers in group 8 on chromosome 2B, one marker in group 10 on chromosome 2B and the marker of group 21 on chromosome 7A. With the exception of the group 7 marker on chromosome 2B, the minor allele frequency decreased from landraces to modern genotypes (registered after 1960) (Figure S2).

## 4. Discussion

The successful interaction of PGPR with plants involves root colonization, the expression of key bacterial genes and the implementation of phytostimulation effects. In this work, the screening of wheat accessions was carried out by focusing on the first two stages, whereas the ecological relevance of the findings was assessed by investigating the third interaction stage, using a subset of four Sp245-stimulating and four non-Sp245-stimulating wheat genotypes. For this screening, the reporter gene *gusA* was preferred to the *egfp* or *mCherry* genes used in other work [44], as the latter were not sensitive enough for the effective quantification of root colonization or *ppdC* expression on the roots. 

The root colonization by *A. baldaniorum* Sp245 varied significantly between replicates, which could be due to its particular root colonization pattern on wheat, making very local cell aggregates, especially near the root tips [14,46,47]. Root colonization also depended on the wheat genotype, with some genotypes displaying ten-fold higher bacterial fluorescence than others, which is reminiscent of findings made with *P. ogarae* F113 [44]. The Spearman’s correlation between fresh root biomass and Sp245(pFAJ31.2) fluorescence intensity was significant (*p* < 0.05, n = 187) but weak (r = 0.24), which suggests that the impact of the root system size on Sp245 colonization was marginal. As for *P. ogarae* F113 [44], the prevalence of ancient genotypes (especially landraces) among the most colonized genotypes was higher than that of modern genotypes, pointing to the adverse effects of modern breeding on PGPR recruitment ability. Importantly, however, the genotypes most (or least) colonized by *A. baldaniorum* Sp245 were largely different from those most (or least) colonized by *P. ogarae* F113, indicating differences in the partnership affinity. 

As in the case of root colonization, *ppdC* expression varied between replicates and between wheat genotypes. IAA biosynthesis is modulated by compounds exudated by roots or present on root cells [15,47,48], whose presence or level may vary depending on the plant genotype [28,32,33,78,79]. This screening can be compared to the one performed with *P. ogarae* F113 and *phl* genes [44], as (i) both DAPG and IAA act as auxinic signals stimulating root system branching and root growth [15,48,80], but (ii) *P. ogarae* F113 is devoid of *ppdC* gene [6] and does not produce IAA. In contrast to *phl* expression, there was no indication that modern breeding had had any negative effect on the ability of the resulting wheat genotypes to stimulate *ppdC* expression. This is consistent with the literature, in that *Azospirillum* strains were shown to readily produce auxins on the roots of modern wheat cultivars, thereby enhancing the number of root hairs and lateral roots [48,49]. 

When considering the implementation of phytostimulation effects by *A. baldaniorum* Sp245 and *P. ogarae* F113, using the same selection of four Sp245/F113 stimulating genotypes and four genotypes stimulating neither Sp245 nor F113 in vitro, it appeared that the occurrence of phytostimulation depended on the interaction between the wheat genotype, PGPR strain and environmental (stress) conditions. Significant phytostimulation was observed for three of the four stimulating genotypes (Coronation and D130-63 for F113, vs. Concurrent for Sp245) and one of the four non-stimulating genotypes (Odesskaya 16 for F113) under the optimum condition, in comparison with all four stimulating genotypes (Concurrent and Coronation for both F113 and Sp245, D130-63 for F113, Amifort for Sp245) and none of the four non-stimulating genotypes under stress [44] (Table S5). Differences in root colonization, gene expression and plant-beneficial effects due to plant genotype have been shown for *P. ogarae* F113 [10,44]. Such differences are also well documented for *A. baldaniorum* Sp245 [81,82] and other *Azospirillum* strains [83,84,85,86,87]. Here, it appeared that the two PGPR displayed different affinity profiles towards the wheat genotypes, which suggests that different genotypes in the same soil may select and rely upon different assortments of PGPR populations.

Twenty-two wheat genomic regions were associated with *A. baldaniorum* Sp245 interaction. However, none of them were identified for root colonization. This may be due to the large variability between replicates, which limited the possibility of detecting small effect associations. Indeed, using the PGPR strain DMS 7030 from the very-closely related species *A. brasilense* and the Opata × synthetic mapping population, Díaz De León et al. [88] were able to detect only one major QTL on chromosome 1A for the physical adhesion of *A. brasilense* cells to wheat roots. QTLs were also sought based on the plant response to *A. brasilense* Ab-V5, but this was conducted in maize [63]. Here, the 22 regions were identified with regard to the expression and/or induction rate of *ppdC* in Sp245. Most of the regions are located on genomes A and B, which is not surprising as it is well-known that wheat genome D is less polymorphic and therefore less covered with SNP markers [68]. In addition, two regions (one on chromosome 2B and the other on 6B) gathered most of the significant markers. This last region comprised the SNP with the largest effect on *ppdC* expression, which could make this region a priority for further genetic studies. Interestingly, nine markers showed different proportions of alleles between the three classes of accessions (landraces, ≤1960, >1960). In eight cases, the frequency of the allele increasing *ppdC* expression was lower in modern cultivars than in landraces. Moreover, although some modern genotypes showed high *ppdC* expression levels, none carried the favorable allele for all the genomic regions identified. QTLs were not sought in the work of Valente et al. [44] with *P. ogarae* F113. When using their raw data for QTL analysis, we found only one significant marker (Table S5), which is another indication pointing to differences in the affinity patterns towards wheat genotypes when considering different types of PGPR strains.

## 5. Conclusions

We found that ancient wheat genotypes were collectively more effective for promoting root colonization and expression of the IAA gene *ppdC* in the PGPR *A. baldaniorum* Sp245 when compared with modern wheat genotypes. The relevance of these findings was shown by the higher Sp245 phytostimulation effects on the PGPR-stimulating wheat genotypes than on the non-PGPR-stimulating genotypes. Importantly, the range of wheat genotypes interacting well with *A. baldaniorum* Sp245 (whether ancient or modern) differed from the range of genotypes interacting well with *P. ogarae* F113, a very different type of PGPR, and the QTL derived from the Sp245 interaction data were not significant for the F113-wheat interaction, indicating that different PGPR interact using different modes of action with a given crop. 

This work identified the first QTL regions associated with molecular interaction of a Poaceae crop with PGPR bacteria. Provided that these genomic regions are not linked to unfavorable alleles for agronomic traits, this opens the possibility to use the molecular markers to increase the frequency of favorable alleles and improve the capacity of modern genotypes to interact with *A. baldaniorum* Sp245, and perhaps also with other *A. baldaniorum* or *Azospirillum* PGPR strains, if the alleles are also relevant at these taxonomic scales.

## Figures and Tables

**Table 1:** Percentages of wheat genotypes showing the highest and lowest values of root colonization by *A. baldaniorum* Sp245(pFAJ31.2) or *ppdC* expression in root-colonizing Sp245(pFAJ64) among the ancient (n = 73) and modern genotypes (n = 114).

	Ancient	Modern
**Root colonization by Sp245**		
25 best genotypes	20.5% a ^†^	8.8% b
50 best genotypes	38.4% a	19.3% b
50 worst genotypes	24.7%	28.1%
25 worst genotypes	13.7%	13.2%
***ppdC* expression in Sp245**		
25 best genotypes	15.1%	12.3%
50 best genotypes	31.5%	23.8%
50 worst genotypes	20.6%	30.7%
25 worst genotypes	6.8% a	17.5% b

^†^ For each row, significant differences between ancient and modern genotypes are indicated by letters a and b (Z-tests carried out on numbers of genotypes, *p* < 0.05).

**Table 2:** Percentages of wheat genotypes showing the highest and lowest values of root colonization (**A**) by *P. ogarae* F113, by *A. baldaniorum* Sp245 or by both PGPR strains among the landraces (n = 23 for most colonized and n = 14 for least colonized), old varieties (≤1960, n = 21 for most colonized and n = 17 for least colonized) and modern genotypes (>1960, n = 42 for most colonized and n = 54 for least colonized), based on the 50 genotypes most or least colonized by F113 and/or the 50 genotypes most or least colonized by Sp245, as well as percentages of wheat genotypes showing the highest and lowest values of gene expression (**B**) in root-colonizing PGPR, when considering *phl* in *P. ogarae* F113, *ppdC* in *A. baldaniorum* Sp245, or both, among the landraces (n = 18 for highest expressions and n = 14 for lowest expressions), old varieties (≤1960, n = 24 for highest expressions and n = 16 for lowest expressions) and modern genotypes (>1960, n = 46 for highest expressions and n = 57 for lowest expressions), based on the 50 genotypes showing the highest or lowest *phl* expression and/or the 50 genotypes showing the highest or lowest *ppdC* expression.

	Landraces	Old Varieties (≤1960)	Modern Genotypes (>1960)
**A** **Genotypes most colonized by Sp245 and/or F113**			
All 86 genotypes	23/23 (100%)	21/21 (100%)	42/42 (100%)
36 genotypes most colonized by Sp245 only	11/23 (47.8%)	7/21 (33.3%)	18/42 (47.6%)
36 genotypes most colonized by F113 only	9/23 (39.1%)	7/21 (33.3%)	20/42 (42.9%)
14 genotypes most colonized by Sp245 and F113	3/23 (13.0%) ab ^†^	7/21 (33.3%) b	4/42 (9.5%) a
**Genotypes least colonized by Sp245 and/or F113**			
All 85 genotypes	14/14 (100%)	17/17 (100%)	54/54 (100%)
35 genotypes least colonized by Sp245 only	8/14 (57.1%)	8/17 (47.1%)	19/54 (35.2%)
35 genotypes least colonized by F113 only	6/14 (42.9%)	7/17 (41.2%)	22/54 (40.7%)
15 genotypes least colonized by Sp245 and F113	0/14 (0%) a	2/17 (11.8%) ab	13/54 (24.1%) b
**B** **Genotypes showing the highest *ppdC* and/or *phl* expression in root-colonizing PGPR**			
All 88 genotypes	18/18 (100%)	24/24 (100%)	46/46 (100%)
38 genotypes with high *ppdC* expression only	7/18 (38.9%)	7/24 (29.2%)	24/46 (52.2%)
38 genotypes with high *phl* expression only	9/18 (50.0%)	10/24 (41.7%)	19/46 (41.3%)
12 genotypes with high *ppdC* and *phl* expression	2/18 (11.1%) ab ^†^	7/24 (29.2%) b	3/46 (6.5%) a
**Genotypes showing the lowest *ppdC* and/or *phl* expression in root-colonizing PGPR**			
All 87 genotypes	14/14 (100%)	16/16 (100%)	57/57 (100%)
37 genotypes with low *ppdC* expression only	4/14 (28.6%)	6/16 (37.5%)	27/57 (47.4%)
37 genotypes with low *phl* expression only	9/14 (64.2%) a	4/16 (25.0%) b	24/57 (42.1%) ab
13 genotypes with low *ppdC* and *phl* expression	1/14 (7.1%) a	6/16 (37.5%) b	6/57 (10.5%) a

^†^ For each row, significant differences between landraces, old varieties and modern genotypes are indicated by letters a and b (Z-tests carried out on numbers of genotypes, *p* < 0.05).

**Table 3:** Chromosomal regions identified after GWAS on 180 bread wheat accessions for *A. baldaniorum* Sp245 colonization, *ppdC* expression and *ppdC* induction rate (considering the means and the medians) with 230,325 SNP markers. For each region, the chromosome assignment, the number of significant markers, the maximum −log_10_(*P*) value, the position of the region on the Chinese Spring RefSeqv1.0 and the PGPR trait concerned are indicated.

Region	Chromosome	Nber Markers	−log_10_(*P*)	End (bp)	Start (bp)	PGPR Trait
1	1A	1	5.4	20,415,992	20,415,992	Induction
2	1B	1	5.0	12,806,570	12,806,570	Expression
3	1B	6	6.5	97,489,862	102,671,353	Expression
4	1B	1	5.6	651,548,215	651,548,215	Expression
5	1B	4	6.2	668,109,643	668,126,415	Expression
6	2A	2	5.2	120,681,877	123,202,999	Expression
7	2B	1	5.1	23,006,356	23,006,356	Expression
8	2B	60	6.8	572,314,013	582,645,061	Expression
9	2B	1	5.7	674,029,409	674,029,409	Induction
10	2B	1	5.2	688,374,113	688,374,113	Expression
11	2B	4	5.2	757,386,253	758,556,708	Induction
12	2B	1	5.4	759,178,940	759,178,940	Induction
13	3A	1	5.7	405,321,848	405,321,848	Expression
14	3A	1	5.1	644,202,467	644,202,467	Expression
15	3B	5	6.5	697,067,059	697,467,601	Induction
16	5B	6	6.7	560,451,114	562,280,657	Expression, Induction
17	6A	4	5.6	51,947,725	51,992,351	Expression, Induction
18	6A	1	5.4	540,840,475	540,840,475	Induction
19	6B	62	6.4	703,207,585	705,314,997	Expression, Induction
20	6B	1	5.2	159,410,401	159,410,401	Induction
21	7A	1	5.1	30,092,884	30,092,884	Expression
22	7D	4	6.0	93,078,495	93,605,114	Expression

## Data Availability

The data that supports the findings of this study are available in the Supplementary Materials of this article.

## Supplementary Materials

The following supporting information can be downloaded at: https://www.mdpi.com/article/10.3390/microorganisms11061615/s1. Reference [89] is cited in the supplementary materials.
