# Supplementary material for: Symbiotic Variations among Wheat Genotypes and Detection of Quantitative Trait Loci for Molecular Interaction with Auxin-Producing Azospirillum PGPR
Source: Microorganisms. 2023 Jun 19;11(6):1615. doi: 10.3390/microorganisms11061615 (PMC10300791; doi:10.3390/microorganisms11061615)
Supplement: Supplementary file 1 [file microorganisms-11-01615-s001.zip › microorganisms-2370575-supplementary.pdf]

Supplementary Information

Symbiotic Variations among Wheat Genotypes and Detection of Quantitative Trait Loci for Molecular Interaction with Auxin-Producing *Azospirillum* PGPR

Jordan Valente [1], Florence Gerin [1], Agathe Mini [2], Rohan Richard [2], Jacques Le Gouis [2], Claire Prigent-Combaret [1] and Yvan Moënne-Loccoz [1,*]

[1] Univ Lyon, Université Claude Bernard Lyon 1, CNRS, INRAE, VetAgro Sup, UMR5557 Ecologie Microbienne, 43 Bd du 11 Novembre 1918, F-69622 Villeurbanne, France

[2] GDEC, INRAE, UCA, F-63000 Clermont-Ferrand, France

Running title: QTL for wheat-PGPR interaction

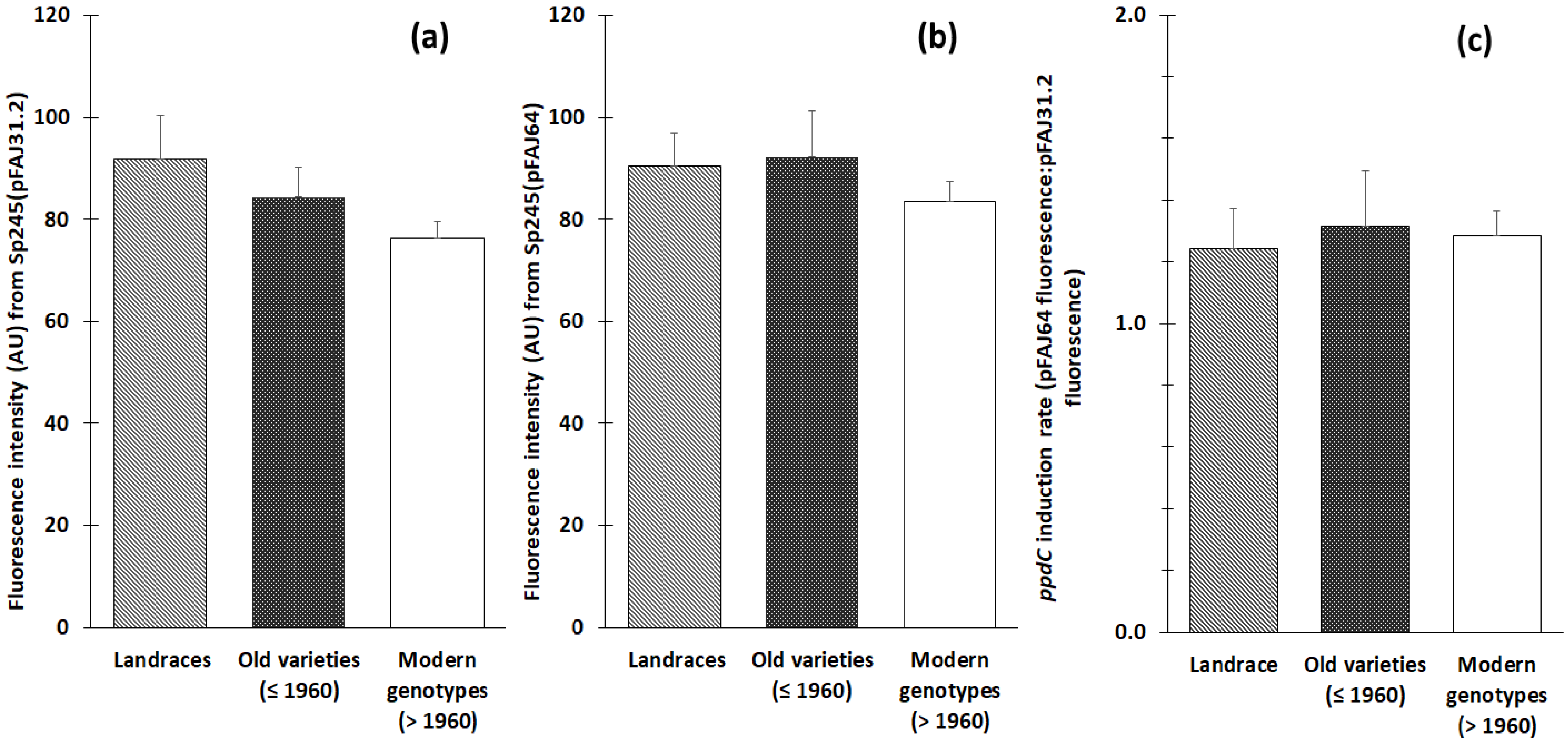

Figure S1. Colonization of *A. baldaniorum* Sp245(pFAJ31.2) and *ppdC* expression in Sp245(pFAJ64) on the roots of landraces (n = 33), old varieties (≤ 1960, n = 40) and modern genotypes (>1960, n = 114). Fluorescence from 4-MU is shown in (a) for the pFAJ31.2 plasmid (root colonization) and in (b) for the pFAJ64 plasmid (*ppdC* expression), whereas the *ppdC* induction rate (pFAJ64 fluorescence:pFAJ31.2 fluorescence ratio) is given in (c). Fluorescence is expressed as arbitrary units (AU) and data are presented as means (computed from individual genotype data) with standard errors. There was no significant difference at $P < 0.05$.

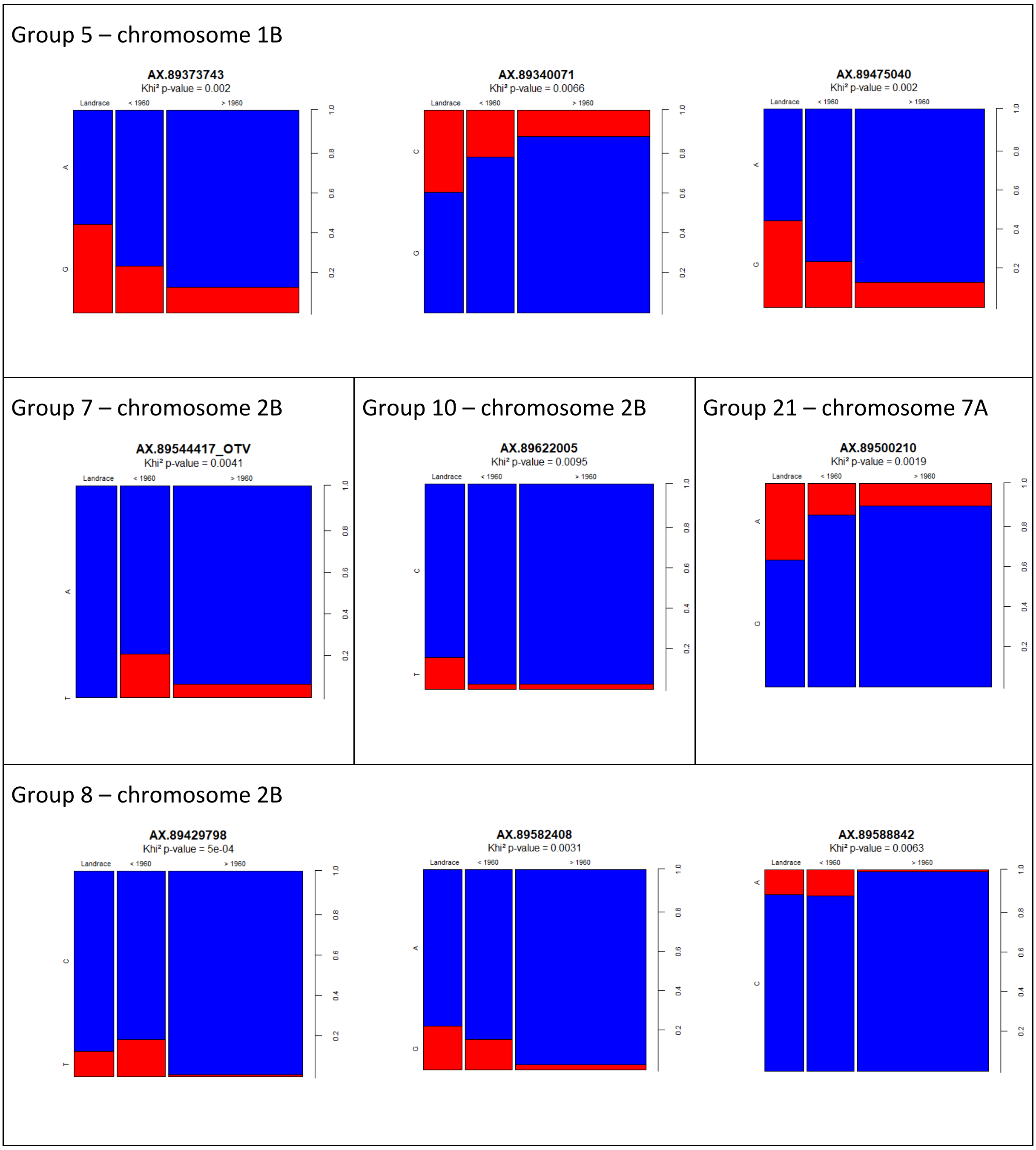

Figure S2. Allele proportion in the three accession classes (landraces, ≤ 1960, > 1960) for the nine markers for which GWAS evidenced a significant difference in allele proportion (Khi^2 tests, $P < 0.01$). Proportions corresponding to the SNP alleles indicated with letters A, G, T or C on the left of each graph are shown in blue for the major allele and in red for the minor allele. Rectangle widths are proportional to accession classes size.

Table S1. List of the 187 wheat genotypes used in the study and their rankings in the screening experiment of three traits: Sp245 colonization, *ppdC* expression and *ppdC* induction rate. These 187 genotypes correspond to the 184 accessions that germinated among the 196 genotypes of the 196CC core-collection sub-sampled from the 372CC collection set up by Balfourier *et al.* (2007)†, plus the reference landrace Chinese Spring and the two reference modern lines Hendrix and Skerzzo. The genotypes are listed according to their ERGE code in the French National Cereal Genetic Resources database. For each of the three rankings done in the screening experiment, the worst rank was 1 and the best rank 187. The total score was obtained by summing the three ranks.

ERGE Code	Genotype	Geographic origin	Genotype category	Sp245 colonization rank	*ppdC* expression rank	*ppdC* induction rate rank	Total score
7	(95-13*BEZOSTAIA)3-3	France	> 1960	55	40	82	177
19	CH01193	Switzerland	> 1960	113	122	110	345
92	11IWSWSN14	USA	> 1960	29	80	143	252
177	DI15	France	> 1960	71	28	51	150
234	DI182-9	France	> 1960	60	158	160	378
236	DI185	France	> 1960	86	60	74	220
338	DI276	France	> 1960	89	185	180	454
347	2838-39	Bulgaria	> 1960	34	71	127	232
386	DI330	France	> 1960	5	38	154	197
419	DI37-12-2	France	> 1960	140	99	60	299
421	3716-1	Bulgaria	> 1960	10	74	164	248
477	DI50-12	France	> 1960	8	146	179	333
524	60293-	The Netherlands	> 1960	109	97	92	298
537	CH62022	Switzerland	> 1960	129	171	140	440
546	664-258-18	Bulgaria	> 1960	83	78	98	259
748	A4	Afghanistan	> 1960	98	128	122	348
794	ADMONTER	Austria	≤ 1960	38	81	125	244
797	ADULAR	Germany	> 1960	148	168	124	440
833	AKADARUMA	Japan	≤ 1960	158	179	134	471
871	ALMA	France	≤ 1960	9	69	162	240
901	AMIFORT	France	> 1960	131	180	156	467
957	ARAWA	Australia or New Zealand	≤ 1960	176	92	16	284
983	ARGENT	UK or Ireland	> 1960	174	113	29	316
1005	ARKAS	Germany	> 1960	178	114	26	318
1032	ARROMANCHES	France	> 1960	102	108	104	314
1044	ARTOIS-DESPREZ	France	≤ 1960	15	95	157	267
1080	ATUT II	Austria	> 1960	159	151	86	396
1192	BALKAN	Croatia	> 1960	45	127	152	324
1232	BARBU DU FINISTERE	France	Landrace	6	118	178	302
1236	BARBU DU TRONCHET	France	Landrace	107	46	44	197
1281	BEL ET BON	France	≤ 1960	121	66	52	239
1288	BELLIEI 590	Hungary	≤ 1960	142	61	38	241
1321	BENNI	USA	> 1960	101	109	106	316
1332	BERZATACA	Finland	> 1960	51	23	66	140
1357	BIRGITTA	Sweden	> 1960	136	54	30	220
1400	BLANC PRECOCE	Switzerland	Landrace	63	104	121	288
1402	BLASON	France	> 1960	78	42	56	176
1417	BLE D'OR	France	≤ 1960	115	30	25	170
1429	BLE DE HAIE	France	Landrace	11	8	107	126
1446	BLE DE MARAT BARBU	France	Landrace	70	133	141	344
1498	BLE DU ROUSSILLON	France	Landrace	151	72	32	255
1529	BLONDYNKA	Poland	≤ 1960	134	111	79	324
1531	BLUEBOY	USA	> 1960	166	148	71	385
1747	114/62	Austria	> 1960	106	27	31	164
1768	CANDEAL DE AREVALO	Spain	Landrace	73	167	165	405

1885	CENAD 512	Romania	≤ 1960	92	149	136	377
1899	CEREALOR	France	> 1960	52	50	95	197
1957	CF3003-2-7-4-4-3	France	> 1960	14	85	155	254
1974	CF4563-1-5-3-2-5	France	> 1960	135	94	58	287
2025	CH73052	Switzerland	> 1960	123	24	14	161
2135	CHINESE SPRING	China	Landrace	141	155	111	407
2145	CHITLANG	Nepal	Landrace	46	160	167	373
2153	CHORTANDINKA	Central Asia	> 1960	120	144	119	383
2169	CHYAKSILA EPI NON VELU	Nepal	Landrace	103	67	72	242
2308	COMPTON	USA	> 1960	108	15	17	140
2345	CORSODOR	France	> 1960	18	2	54	74
2364	CP4	France	> 1960	85	62	78	225
2399	D130-63	Poland	> 1960	97	166	153	416
2424	DANUBIA	Czech Republic	> 1960	104	18	23	145
2438	DAVIDOC	France	> 1960	26	77	144	247
2475	DETENICKA CERVENA	Czech Republic	Landrace	68	125	135	328
2489	DI6402-34-2-4	France	> 1960	67	98	117	282
2491	DI6404-19-15	France	> 1960	36	33	100	169
2507	DI7003-1-12	France	> 1960	112	25	24	161
2508	DI7005-113-3	France	> 1960	59	124	139	322
2526	DI7202-103	France	> 1960	77	184	182	443
2534	DI7210-15-11	France	> 1960	53	131	151	335
2536	DI7215-100	France	> 1960	186	136	12	334
2573	DIANA	Poland	> 1960	35	186	186	407
2574	DIANA II	Czech Republic	> 1960	105	82	87	274
2606	DNEPROVSKAIA	Ukraine	> 1960	21	83	150	254
2626	DONG-FANG-HONG-NO3	China	> 1960	1	93	181	275
2644	DRAGON-FRA	France	> 1960	3	130	183	316
2650	DRAVA	Croatia	> 1960	137	139	103	379
2683	E108	France	≤ 1960	ND	ND	ND	ND
2698	EBRO	Spain	> 1960	173	132	48	353
2802	ESPOIR	France	≤ 1960	170	178	118	466
2991	FERRUGINEUM	Russia	≤ 1960	44	31	83	158
3050	FLAMURA 85	Romania	> 1960	114	70	67	251
3070	FLINT	USA	Landrace	163	119	47	329
3278	GELPA	France	> 1960	87	84	96	267
3299	GH126	France	> 1960	65	9	37	111
3342	GK SZOKE	Hungary	> 1960	110	73	73	256
3366	GODOLLOI 15	Hungary	≤ 1960	ND	ND	ND	ND
3406	GRANIT	Russia	> 1960	155	58	18	231
3414	GRENIER	France	> 1960	50	170	173	393
3485	H93-70	Spain	> 1960	4	164	185	353
3617	HIVERNAL	France	> 1960	43	103	133	279
3753	IAS 1	Brazil	≤ 1960	96	110	108	314
3896	JANGO	France	≤ 1960	7	53	158	218
3912	JASZSAGI TF	Hungary	≤ 1960	25	12	85	122
3970	JUFY II	Belgium	≤ 1960	91	65	76	232
3991	K1898-9/L200-6	Bulgaria	> 1960	172	140	55	367
4036	KATYIL	Australia or New Zealand	> 1960	19	3	69	91
4105	KID	France	> 1960	12	11	101	124
4111	KIRAC 66	Turkey	> 1960	122	64	50	236
4157	KOLBEN 3	Sweden	Landrace	ND	ND	ND	ND
4187	KRAKA	Norway or Denmark	> 1960	145	176	142	463
4194	KRELOF 3	France	≤ 1960	133	152	114	399
4300	LESZYNSKA WCZESNA	Poland	≤ 1960	150	76	41	267
4324	LITTLE CLUB	USA	Landrace	69	52	77	198
4343	LONTOI	Finland	> 1960	168	32	6	206
4525	MALGORZATKA UDYCKA	Poland	≤ 1960	119	26	19	164

4664	MASTER	UK or Ireland	> 1960	144	121	81	346
4670	MATRADERECSKEITF	Hungary	> 1960	54	22	61	137
4838	MINTURK	USA	≤ 1960	100	157	137	394
4947	MOTTIN	France	Landrace	147	161	113	421
4991	MV MA	Hungary	> 1960	127	172	145	444
5293	NOUGAT	France	> 1960	28	162	177	367
5401	NZ(81)P43	Australia or New Zealand	> 1960	185	165	45	395
5421	ODESSA EXPSTA20722	Portugal	> 1960	62	10	40	112
5438	ODESSKAYA 16	Ukraine	≤ 1960	23	7	80	110
5448	OGOSTA	Bulgaria	> 1960	132	90	62	284
5501	ORLANDI	Italy	≤ 1960	13	5	93	111
5536	OULIANOWSKA	Russia	> 1960	116	29	22	167
5552	P. DE BROLLON	Spain	Landrace	22	105	159	286
5558	P4523-80	Austria	> 1960	124	138	112	374
5773	POILU DU TARN	France	≤ 1960	61	37	70	168
6027	RECITAL	France	> 1960	32	20	88	140
6086	RENAN	France	> 1960	99	182	171	452
6191	RINGOT 2	France	≤ 1960	2	141	184	327
6308	ROUGE D'ALTKIRCH	France	Landrace	125	79	63	267
6318	ROUGE DE MARCHISSY	Switzerland	Landrace	33	153	172	358
6529	SEU SEUN 27	China	≤ 1960	157	175	129	461
6740	STRUBES DICKKOPF	Germany	≤ 1960	181	100	13	294
6922	TF6	France	≤ 1960	139	101	65	305
6986	TOM THUMB	USA	> 1960	66	117	130	313
7011	TOUZELLE-BLANCHE-BARBUE	France	Landrace	93	143	132	368
7085	TURDA 81-77	Romania	> 1960	161	96	27	284
7092	TYLER	USA	> 1960	20	35	115	170
7117	US(59)34	USA	> 1960	24	44	116	184
7166	US(62)P66	Colombia	> 1960	ND	ND	ND	ND
7279	VALDOR	France	≤ 1960	58	173	174	405
7490	VPM V1-1-2-4R2-3-8-3-2	France	> 1960	81	102	109	292
7585	WATTINES	France	> 1960	153	112	57	322
7848	RONGOTEA	Australia or New Zealand	> 1960	31	89	146	266
7968	BLE DANOIS	France	Landrace	30	135	170	335
7973	BORDEAUX 113	France	Landrace	47	48	97	192
7988	CREPIN A	France	≤ 1960	165	86	21	272
8011	INSTITUT 1802	France	≤ 1960	57	36	75	168
8048	RALET	France	Landrace	183	41	4	228
8051	BLE BARBU DE MUROL	France	Landrace	162	116	46	324
8058	ZANDA	Belgium	≤ 1960	160	154	89	403
8073	CORONATION	Canada	≤ 1960	72	169	168	409
8079	KITCHENER	Canada	≤ 1960	88	150	138	376
8097	STANLEY	Canada	≤ 1960	179	123	36	338
8165	NAVARRO150	Spain	> 1960	40	120	149	309
8170	WS-13 CARDENO 34/45	Spain	> 1960	ND	ND	ND	ND
8194	NEELKANT	Syria	> 1960	64	134	147	345
8197	SANUNU	Syria	> 1960	76	47	68	191
8227	NISHIKAZE KOMUGI	Japan	> 1960	39	156	169	364
8254	CADENZA	France	> 1960	143	137	99	379
8276	CARIBO	Germany	> 1960	17	56	131	204
8287	DC147U	France	> 1960	42	142	163	347
8289	TM7MB1-1	France	> 1960	74	16	39	129
9024	GENESIS	France	> 1960	ND	ND	ND	ND
9077	NON PLUS EXTRA	Austria	Landrace	187	51	2	240
9087	PRINCE LEOPOLD	Belgium	≤ 1960	154	174	126	454
13210	SOLARIS	Czech Republic	> 1960	49	57	105	211
13282	ANATOLIE2	France	≤ 1960	177	63	9	249
13292	CONCURRENT	France	≤ 1960	182	145	43	370

13310	FRUH-WEIZEN	Germany	Landrace	ND	ND	ND	ND
13436	FONDARD CRESPIN	France	≤ 1960	94	34	42	170
13445	VOLT	Hungary	> 1960	16	147	176	339
13454	SPONSOR	France	> 1960	ND	ND	ND	ND
13461	BEHERT	France	> 1960	95	21	28	144
13471	ORNICAR	France	> 1960	184	4	1	189
13481	APACHE	France	> 1960	128	181	161	470
13494	BELLOVAC	France	> 1960	79	91	102	272
13500	ORFIELD	France	> 1960	130	39	20	189
13502	PALIO	France	> 1960	27	13	84	124
13792	CENTURK	USA	> 1960	37	106	148	291
13861	AUGUSTE	France	> 1960	117	159	128	404
13870	TALISMAN	France	> 1960	111	59	59	229
14000	ROKYCANSKA SAMETKA	Czech Republic	Landrace	48	88	123	259
14011	HANA	Czech Republic	> 1960	90	75	90	255
15606	BLE DE REDON BLANC BARBU 1 1	France	Landrace	156	55	15	226
15658	BLE DE REDON BLANC 1/2 LACHE 1 1	France	Landrace	118	183	175	476
15710	BLE DE REDON GLUMES VELUES 1	France	Landrace	146	129	91	366
15950	AS68VM4-3-2/TJB636 13	France	> 1960	82	19	35	136
15954	ASVM4/BEAUCHAMP 81B13	France	> 1960	126	68	49	243
20074	MIRLEBEN	Ukraine	> 1960	164	6	3	173
20224	FANTASIYA-ODESSKAYA	Ukraine	> 1960	ND	ND	ND	ND
20276	EQUINOX	UK or Ireland	> 1960	152	45	11	208
20366	SKERZZO	France	> 1960	149	14	7	170
20384	DI9234-11-15	France	> 1960	75	1	8	84
20417	HAMAC	The Netherlands	> 1960	ND	ND	ND	ND
24031	KRASNAYA	Canada	Landrace	180	87	10	277
24058	SARI-BUGDA	Caucasia	Landrace	175	115	33	323
24066	CROISEMENT 268	Switzerland	≤ 1960	80	43	53	176
24075	SPIN, 121-VAR.12/536	Pakistan	≤ 1960	41	187	187	415
24089	TAU-BUGDA	Caucasia	Landrace	167	107	34	308
24108	ALBIDUM 12	Russia	> 1960	138	126	94	358
24193	LANDRACE	Caucasia	Landrace	171	17	5	193
24196	ARABUGDASI	Caucasia	Landrace	169	177	120	466
24210	LAMMAS	UK or Ireland	Landrace	56	163	166	385
28978	HENDRIX	France	> 1960	84	49	64	197

† **Balfourier F, Roussel V, Strelchenko P, Exbrayat-Vinson F, Sourdille P, Boutet G *et al.*** 2007. A worldwide bread wheat core collection arrayed in a 384-well plate. Theoretical and Applied Genetics **114**, 1265–1275.

Table S2. Percentages of wheat genotypes showing the highest and lowest values of root colonization by *A. baldaniorum* Sp245 or *ppdC* expression in root-colonizing Sp245 among the landraces (n = 33), old varieties (≤ 1960, n = 40) and modern genotypes (>1960, n = 114).

	Landraces	Old varieties (≤ 1960)	Modern genotypes (> 1960)
Root colonization by Sp245			
25 best genotypes	24.2 %	17.5 %	8.8 %
50 best genotypes	42.4 % a†	35.0 % ab	19.3 % b
50 worst genotypes	24.2 %	25.0 %	28.1 %
25 worst genotypes	9.1 %	17.5 %	13.2 %
ppdC expression in Sp245			
25 best genotypes	12.1 %	17.5 %	12.3 %
50 best genotypes	27.3 %	35.0 %	23.8 %
50 worst genotypes	15.2 %	25.0 %	30.7 %
25 worst genotypes	6.1 % a	7.5 %	17.5 %

†For each row, significant differences between landraces, old varieties and modern genotypes are indicated by letters a and b (χ^2 tests carried out on numbers of genotypes, $P < 0.05$).

Table S3. Relative impact of combined stress on the growth of eight non-inoculated wheat genotypes. Concurrent, Coronation, Amifort and D130-63 are Sp245-stimulating genotypes, Jaszaji TF, Odesskaya 16, Danubia and Hendrix are non Sp245-stimulating genotypes. For each of the eight plant parameters investigated, the relative impacts were computed as (stress – optimum)/optimum. For each genotype, significant differences between stressed plants and controls are indicated in bold. Statistical level is indicated with * (*P* < 0.05), ** (*P* < 0.01) or *** (*P* < 0.001), based on ANOVA and Fisher's LSD tests on raw plant values.

	Root volume (cm^3)	Fresh root biomass (mg)	Dry root biomass (mg)	Number of roots	Root average diameter (mm)	Root length (cm)	Fresh shoot biomass (mg)	Dry shoot biomass (mg)
Concurrent	-32.4%	-32.5%	-22.5%	22.6%	**-10.3%***	20.2%	**-48.8% ***	**-49.4% ***
Coronation	-24.6%	-29.1%	-24.5%	19.7%	**-20.1% ***	-16.4%	**-36.8% ***	-31.3%
Amifort	-20.0%	-16.5%	-21.9%	4.5%	**-19.3% ***	-1.3%	**-54.7% ***	**-56.6% ***
D130-63	**-39.0% ***	-26.5%	-16.4%	4.0%	8.1%	17.6%	**-49.4% ***	**-43.3% ***
Jaszaji TF	**-47.1% ***	-25.3%	-18.6%	-8.2%	**-23.7% ***	2.1%	**-47.9% ***	**-45.1% ***
Odesskaya 16	-22.9%	-13.7%	-26.1%	2.3%	2.2%	25.4%	**-40.1% ***	**-42.3% ***
Danubia	**-54.8% ***	**-43.7% ***	-17.4%	-13.1%	**-13.4% ***	-17.6%	**-42.3% ***	**-43.6% ***
Hendrix	**-59.0% ***	**-55.1% ***	**-59.2% ***	-6.1%	**-15.5% ***	**-37.3%***	-19.92%	-16.7%

Table S4. Relative impact of seed inoculation with *A. baldaniorum* Sp245 on the growth of eight wheat genotypes under optimum or combined stress conditions. Concurrent, Coronation, Amifort and D130-63 are Sp245-stimulating genotypes, Jaszaji TF, Odesskaya 16, Danubia and Hendrix are non Sp245-stimulating genotypes. For each of the eight plant parameters investigated, the relative impacts were computed as (inoculated – non-inoculated)/non-inoculated. For each genotype, significant differences between inoculated plants and controls are indicated in bold. Statistical level is indicated with * (*P* < 0.05), ** (*P* < 0.01) or *** (*P* < 0.001), based on ANOVA and Fisher's LSD tests on raw plant values.

		Root volume (cm^3)	Fresh root biomass (mg)	Dry root biomass (mg)	Number of roots	Root average diameter (mm)	Root length (cm)	Fresh shoot biomass (mg)	Dry shoot biomass (mg)
Optimum	Concurrent	61.9%	32.4%	**38.9% ***	**94.6% ****	4.9%	12.0%	2.9%	3.5%
	Coronation	10.3%	10.1%	8.6%	21.1%	2.5%	1.9%	8.0%	4.4%
	Amifort	37.2%	16.6%	16.6%	48.6%	-0.5%	4.7%	1.0%	-4.4%
	D130-63	-1.7%	3.2%	6.9%	0.8%	1.5%	4.3%	14.8%	9.9%
	Jaszaji TF	4.3%	3.0%	-4.4%	1.1%	-4.0%	8.4%	2.1%	-3.8%
	Odesskaya 16	18.6%	17.8%	16.6%	21.3%	2.9%	4.0%	-2.4%	-1.0%
	Danubia	14.4%	7.7%	10.8%	11.6%	2.3%	4.0%	8.6%	10.2%
	Hendrix	5.5%	1.6%	-6.2%	10.2%	-2.3%	9.8%	0.0%	-4.9%
Stress	Concurrent	**75.8% ***	**54.2% ****	**57.0% ****	**80.6% *****	-2.5%	15.4%	9.2%	9.9%
	Coronation	38.4%	19.9%	25.9%	**88.3% *****	2.1%	8.8%	13.5%	15.6%
	Amifort	**49.6% ***	**43.2% ****	**52.2% ****	**86.3% *****	7.9%	14.5%	6.4%	6.3%
	D130-63	7.3%	9.6%	6.8%	6.4%	-5.4%	5.1%	6.9%	16.1%
	Jaszaji TF	12.4%	13.7%	7.5%	11.1%	0.2%	1.1%	-5.8%	0.6%
	Odesskaya 16	17.8%	17.8%	21.5%	37.4%	-3.5%	1.9%	5.9%	9.3%
	Danubia	12.2%	10.7%	10.2%	18.9%	2.4%	-14.9%	2.4%	3.1%
	Hendrix	4.8%	3.2%	7.1%	10.2%	5.0%	-3.3%	-1.1%	1.7%

Table S5. Detection of genetic markers in wheat genome involved in interactions with *A. baldaniorum* Sp245†. The group number identifies the QTL where the markers are located. The trait indicates the type of result linked to the genetic markers, i.e. bacterial strain name (Sp245) plus bacterial trait (Exp for *ppdC* expression and Ind for *ppdC* induction rate) plus metrics (Moy for mean and Med for median). The marker designates the name of the SNP. The position corresponds to the physical position of the markers on wheat chromosomes (based on Chinese Spring RefSeqv1.0). A and B stand for major allele and minor allele, respectively. NoMeasured indicates the number of accessions measured. NbA is the number of accessions with the major allele and NbB the number of accessions with the minor allele. EffB indicates the effect of the minor allele on the trait and SE_effB the standard error of the effect of the minor allele. p_Marker is the *P* value and -log10(*P*) the positive log value. Khi² was used to test the hypothesis that the three accessions classes (landraces, ≤ 1960, > 1960) have the same marker allele proportions, and highly significant ($P < 0.01$) differences (found on nine occasions) are indicated in bold.

Group	Trait	Marker	Chromosome	Position	A	B	NoMeasured	NbA	NbB	effB	SE_effB	p_Marker	$-\log_{10}(P)$	Khi²
1	Sp245.Ind.Moy	AX.89366874_OTV	1A	20415992	A	T	174	164	10	0.91	0.1978	4.10E-06	5.39	0.0127
2	Sp245.Exp.Med	AX.89451818	1B	12806570	T	A	175	145	30	20.66	4.6487	8.82E-06	5.05	0.0666
3	Sp245.Exp.Med	AX.89653443	1B	97489862	G	A	176	162	14	28.98	6.4564	7.16E-06	5.14	0.0714
3	Sp245.Exp.Med	AX.89652072	1B	97619942	T	A	176	162	14	28.98	6.4564	7.16E-06	5.14	0.0714
3	Sp245.Exp.Med	AX.89346256	1B	99959384	C	T	176	164	12	33.94	6.9310	9.75E-07	6.01	0.0462
3	Sp245.Exp.Med	AX.89335073	1B	100561973	C	A	175	163	12	35.33	6.9325	3.47E-07	6.46	0.1887
3	Sp245.Exp.Med	AX.89367916	1B	102627427	T	G	176	163	13	32.14	6.6795	1.49E-06	5.83	0.0906
3	Sp245.Exp.Med	AX.89496064	1B	102671353	A	G	176	163	13	32.14	6.6795	1.49E-06	5.83	0.0906
4	Sp245.Exp.Med	AX.89462719	1B	651548215	C	T	176	167	9	37.38	7.9310	2.43E-06	5.61	0.1020
5	Sp245.Exp.Med	AX.89373743	1B	668109643	A	G	176	141	35	21.08	4.3769	1.46E-06	5.84	**0.0020**
5	Sp245.Exp.Med	AX.89415492	1B	668122408	G	C	176	144	32	20.92	4.5295	3.87E-06	5.41	0.0117
5	Sp245.Exp.Med	AX.89340071	1B	668125848	G	C	176	142	34	22.08	4.4251	6.05E-07	6.22	**0.0066**
5	Sp245.Exp.Med	AX.89475040	1B	668126415	A	G	176	141	35	21.08	4.3769	1.46E-06	5.84	**0.0020**
6	Sp245.Exp.Med	AX.89715338	2A	120681877	C	T	176	168	8	37.88	8.3871	6.30E-06	5.20	0.9160
6	Sp245.Exp.Med	AX.89757505	2A	123202999	A	G	176	167	9	35.28	7.9310	8.66E-06	5.06	0.6496
7	Sp245.Exp.Med	AX.89544417_OTV	2B	23006356	A	T	176	161	15	28.08	6.2568	7.20E-06	5.14	**0.0041**
8	Sp245.Exp.Med	AX.89701067	2B	572314013	T	C	176	136	40	20.09	4.1688	1.44E-06	5.84	0.0632
8	Sp245.Exp.Med	AX.89558788	2B	572321667	C	T	176	166	10	36.92	7.5467	9.96E-07	6.00	0.0213
8	Sp245.Exp.Med	AX.89588842	2B	572557343	C	A	176	167	9	40.17	7.9310	4.08E-07	6.39	**0.0063**
8	Sp245.Exp.Med	AX.89608961	2B	572586442	C	T	176	167	9	36.15	7.9310	5.18E-06	5.29	0.0245
8	Sp245.Exp.Med	AX.89400900	2B	572622617	C	T	176	166	10	36.92	7.5467	9.96E-07	6.00	0.0213
8	Sp245.Exp.Med	AX.89735345	2B	572670087	C	A	176	167	9	41.17	7.9310	2.09E-07	6.68	0.0245

8	Sp245.Exp.Med	AX.89472541	2B	572789475	T	C	176	166	10	36.92	7.5467	9.96E-07	6.00	0.0213
8	Sp245.Exp.Med	AX.89625338	2B	573052357	A	T	176	166	10	36.92	7.5467	9.96E-07	6.00	0.0213
8	Sp245.Exp.Med	AX.89582408	2B	573199626	A	G	176	161	15	30.38	6.2568	1.20E-06	5.92	**0.0031**
8	Sp245.Exp.Med	AX.89656849	2B	573203394	A	G	176	167	9	41.17	7.9310	2.09E-07	6.68	0.0245
8	Sp245.Exp.Med	AX.89451317	2B	573205653	C	T	176	128	48	17.41	3.9227	9.11E-06	5.04	0.0461
8	Sp245.Exp.Med	AX.89474485	2B	573205674	C	T	176	128	48	17.41	3.9227	9.11E-06	5.04	0.0461
8	Sp245.Exp.Med	AX.89716141	2B	573205718	T	C	176	166	10	36.92	7.5467	9.96E-07	6.00	0.0213
8	Sp245.Exp.Med	AX.89546023	2B	573207779	T	G	176	128	48	17.41	3.9227	9.11E-06	5.04	0.0461
8	Sp245.Exp.Med	AX.89435742	2B	573210497	C	T	176	128	48	17.41	3.9227	9.11E-06	5.04	0.0461
8	Sp245.Exp.Med	AX.89604763	2B	573219613	G	A	176	166	10	36.92	7.5467	9.96E-07	6.00	0.0213
8	Sp245.Exp.Med	AX.89392471	2B	573593898	C	G	176	166	10	36.92	7.5467	9.96E-07	6.00	0.0213
8	Sp245.Exp.Med	AX.89536611	2B	573672989	C	A	176	166	10	36.92	7.5467	9.96E-07	6.00	0.0213
8	Sp245.Exp.Med	AX.89508647	2B	573688461	T	C	174	164	10	36.82	7.5493	1.08E-06	5.97	0.0235
8	Sp245.Exp.Med	AX.89712625	2B	573688492	C	A	176	166	10	36.92	7.5467	9.96E-07	6.00	0.0213
8	Sp245.Exp.Med	AX.89543231	2B	573720522	C	T	176	166	10	36.92	7.5467	9.96E-07	6.00	0.0213
8	Sp245.Exp.Med	AX.89576079	2B	574041955	T	C	176	129	47	18.15	3.9488	4.27E-06	5.37	0.0312
8	Sp245.Exp.Med	AX.89635740	2B	574042114	G	T	176	166	10	36.92	7.5467	9.96E-07	6.00	0.0213
8	Sp245.Exp.Med	AX.89386011	2B	574043333	G	C	176	166	10	36.92	7.5467	9.96E-07	6.00	0.0213
8	Sp245.Exp.Med	AX.89345560	2B	574693828	T	C	176	127	49	17.38	3.8977	8.19E-06	5.09	0.0662
8	Sp245.Exp.Med	AX.89388511	2B	574709328	G	A	176	127	49	17.38	3.8977	8.19E-06	5.09	0.0662
8	Sp245.Exp.Med	AX.89338777	2B	574710599	A	G	176	127	49	17.38	3.8977	8.19E-06	5.09	0.0662
8	Sp245.Exp.Med	AX.89764839	2B	574712159	C	A	176	166	10	36.92	7.5467	9.96E-07	6.00	0.0213
8	Sp245.Exp.Med	AX.89584393	2B	574712834	A	G	176	127	49	17.38	3.8977	8.19E-06	5.09	0.0662
8	Sp245.Exp.Med	AX.89417406	2B	574713427	C	T	176	127	49	17.38	3.8977	8.19E-06	5.09	0.0662
8	Sp245.Exp.Med	AX.89479386	2B	574713488	C	T	176	166	10	36.92	7.5467	9.96E-07	6.00	0.0213
8	Sp245.Exp.Med	AX.89477474	2B	574715971	A	G	176	127	49	17.38	3.8977	8.19E-06	5.09	0.0662
8	Sp245.Exp.Med	AX.89464190	2B	574716544	G	A	176	167	9	36.15	7.9310	5.18E-06	5.29	0.0245
8	Sp245.Exp.Med	AX.89626581	2B	575115755	C	A	176	127	49	17.38	3.8977	8.19E-06	5.09	0.0662
8	Sp245.Exp.Med	AX.89416120	2B	575147373	A	G	176	130	46	18.39	3.9761	3.76E-06	5.42	0.0205
8	Sp245.Exp.Med	AX.89465706	2B	575147405	C	T	176	166	10	36.92	7.5467	9.96E-07	6.00	0.0213
8	Sp245.Exp.Med	AX.89384765	2B	575147497	T	A	176	166	10	36.92	7.5467	9.96E-07	6.00	0.0213

8	Sp245.Exp.Med	AX.89701142	2B	575148227	A	G	176	130	46	18.39	3.9761	3.76E-06	5.42	0.0205
8	Sp245.Exp.Med	AX.89589714	2B	575149606	A	T	176	127	49	17.38	3.8977	8.19E-06	5.09	0.0662
8	Sp245.Exp.Med	AX.89343018	2B	575149623	T	C	176	127	49	17.38	3.8977	8.19E-06	5.09	0.0662
8	Sp245.Exp.Med	AX.89374062	2B	575165223	A	G	176	130	46	18.39	3.9761	3.76E-06	5.42	0.0205
8	Sp245.Exp.Med	AX.89426922	2B	575171812	A	G	176	127	49	17.38	3.8977	8.19E-06	5.09	0.0662
8	Sp245.Exp.Med	AX.89419933	2B	575174058	C	T	173	129	44	18.83	4.0463	3.27E-06	5.49	0.0320
8	Sp245.Exp.Med	AX.89743901	2B	575176667	G	C	176	127	49	17.38	3.8977	8.19E-06	5.09	0.0662
8	Sp245.Exp.Med	AX.89429798	2B	575235683	C	T	176	164	12	33.53	6.9310	1.31E-06	5.88	**0.0005**
8	Sp245.Exp.Med	AX.89434650	2B	575339365	C	T	176	167	9	41.75	7.9310	1.41E-07	6.85	0.0171
8	Sp245.Exp.Med	AX.89351473	2B	575377838	T	C	176	127	49	17.38	3.8977	8.19E-06	5.09	0.0662
8	Sp245.Exp.Med	AX.89593657	2B	575385248	A	G	176	127	49	17.38	3.8977	8.19E-06	5.09	0.0662
8	Sp245.Exp.Med	AX.89387691	2B	575706535	G	C	176	166	10	36.92	7.5467	9.96E-07	6.00	0.0213
8	Sp245.Exp.Med	AX.89457236	2B	575706596	C	T	176	166	10	36.92	7.5467	9.96E-07	6.00	0.0213
8	Sp245.Exp.Med	AX.89521523	2B	575706667	C	T	175	165	10	36.95	7.5480	9.84E-07	6.01	0.0224
8	Sp245.Exp.Med	AX.89335163	2B	575837447	G	A	174	138	36	22.74	4.3375	1.58E-07	6.80	0.0224
8	Sp245.Exp.Med	AX.89746318	2B	575864365	A	G	176	136	40	18.82	4.1688	6.34E-06	5.20	0.0399
8	Sp245.Exp.Med	AX.89385117	2B	575865205	T	C	175	135	40	18.68	4.1723	7.55E-06	5.12	0.0432
8	Sp245.Exp.Med	AX.89719394	2B	575877955	T	G	176	127	49	17.86	3.8977	4.62E-06	5.34	0.0998
8	Sp245.Exp.Med	AX.89472819	2B	575930503	C	G	176	130	46	17.84	3.9761	7.27E-06	5.14	0.0337
8	Sp245.Exp.Med	AX.89698767	2B	575933438	T	C	176	153	23	24.44	5.1832	2.41E-06	5.62	0.0912
8	Sp245.Exp.Med	AX.89540301	2B	575934051	C	T	173	127	46	19.04	3.9884	1.81E-06	5.74	0.0188
8	Sp245.Exp.Med	AX.89497267	2B	581582603	T	C	176	163	13	30.71	6.6795	4.27E-06	5.37	0.5379
8	Sp245.Exp.Med	AX.89711832	2B	582645061	G	A	176	164	12	32.61	6.9310	2.55E-06	5.59	0.3777
9	Sp245.Ind.Moy	AX.89668550	2B	674029409	G	T	174	165	9	0.99	0.2079	2.00E-06	5.70	0.6496
10	Sp245.Exp.Med	AX.89622005	2B	688374113	C	T	176	168	8	37.88	8.3871	6.30E-06	5.20	**0.0095**
11	Sp245.Ind.Moy	AX.89511582	2B	757386253	T	C	174	162	12	0.81	0.1817	7.85E-06	5.10	0.8877
11	Sp245.Ind.Moy	AX.89431594	2B	757452859	C	T	174	163	11	0.86	0.1892	5.71E-06	5.24	0.9574
11	Sp245.Ind.Moy	AX.89441916	2B	758112233	C	T	174	163	11	0.86	0.1892	5.71E-06	5.24	0.9574
11	Sp245.Ind.Moy	AX.89526336	2B	758556708	G	A	174	163	11	0.86	0.1892	5.71E-06	5.24	0.9574
12	Sp245.Ind.Moy	AX.89757265	2B	759178940	C	T	174	162	12	0.84	0.1817	3.73E-06	5.43	0.2448
13	Sp245.Exp.Med	AX.89467602	3A	405321848	A	G	176	152	24	24.26	5.0908	1.88E-06	5.73	0.0218

14	Sp245.Exp.Med	AX.89352119	3A	644202467	A	G	176	161	15	27.85	6.2568	8.52E-06	5.07	0.6946
15	Sp245.Ind.Moy	AX.89343547	3B	697067059	G	A	174	166	8	1.12	0.2199	3.48E-07	6.46	0.8638
15	Sp245.Ind.Moy	AX.89648213	3B	697094433	G	A	174	166	8	1.12	0.2199	3.48E-07	6.46	0.8638
15	Sp245.Ind.Moy	AX.89536337	3B	697094560	G	C	174	166	8	1.12	0.2199	3.48E-07	6.46	0.8638
15	Sp245.Ind.Moy	AX.89548989	3B	697097583	C	A	174	166	8	1.12	0.2199	3.48E-07	6.46	0.8638
15	Sp245.Ind.Moy	AX.89710913	3B	697467601	A	G	174	166	8	1.12	0.2199	3.48E-07	6.46	0.8638
16	Sp245.Ind.Med	AX.89467143	5B	560451114	T	C	174	157	17	0.52	0.1164	7.97E-06	5.10	0.5848
16	Sp245.Exp.Med	AX.89625435	5B	560510249	C	T	176	160	16	27.76	6.0770	4.91E-06	5.31	0.5118
16	Sp245.Ind.Med	AX.89625435	5B	560510249	C	T	174	158	16	0.56	0.1196	2.38E-06	5.62	0.5118
16	Sp245.Exp.Med	AX.89746797	5B	560744181	G	A	176	162	14	33.62	6.4564	1.92E-07	6.72	0.9718
16	Sp245.Ind.Med	AX.89746797	5B	560744181	G	A	174	160	14	0.66	0.1271	1.94E-07	6.71	0.9718
16	Sp245.Ind.Med	AX.89525713	5B	562280657	G	C	174	151	23	0.47	0.1021	4.31E-06	5.37	0.7741
17	Sp245.Exp.Med	AX.89488865	6A	51947725	C	T	176	142	34	19.58	4.4251	9.70E-06	5.01	0.8022
17	Sp245.Ind.Med	AX.89488865	6A	51947725	C	T	174	142	32	0.40	0.0892	7.23E-06	5.14	0.8022
17	Sp245.Exp.Med	AX.89734201	6A	51951424	C	A	176	157	19	26.27	5.6297	3.06E-06	5.51	0.0394
17	Sp245.Exp.Med	AX.89673138	6A	51992351	A	G	176	156	20	25.90	5.5047	2.54E-06	5.60	0.0233
18	Sp245.Ind.Moy	AX.89586813	6A	540840475	C	T	174	162	12	0.84	0.1817	3.74E-06	5.43	0.4327
19	Sp245.Ind.Moy	AX.89668441	6B	703207585	G	A	174	161	13	0.83	0.1751	2.44E-06	5.61	0.5146
19	Sp245.Ind.Moy	AX.89490161	6B	703218204	T	C	174	160	14	0.77	0.1693	6.18E-06	5.21	0.5158
19	Sp245.Ind.Moy	AX.89745923	6B	703285156	G	A	174	160	14	0.77	0.1693	6.18E-06	5.21	0.5158
19	Sp245.Exp.Med	AX.89635801	6B	703286795	G	T	176	161	15	30.37	6.2568	1.21E-06	5.92	0.9284
19	Sp245.Ind.Moy	AX.89393793	6B	703298743	C	T	174	160	14	0.77	0.1693	6.18E-06	5.21	0.5158
19	Sp245.Ind.Moy	AX.89471662	6B	703834232	C	T	174	160	14	0.77	0.1693	6.18E-06	5.21	0.5158
19	Sp245.Exp.Med	AX.89585079	6B	704154311	C	T	176	156	20	24.87	5.5047	6.25E-06	5.20	0.2370
19	Sp245.Ind.Moy	AX.89739667	6B	704154523	G	A	174	160	14	0.77	0.1693	6.18E-06	5.21	0.5158
19	Sp245.Exp.Med	AX.89580771	6B	704176383	A	G	176	161	15	29.07	6.2568	3.39E-06	5.47	0.8336
19	Sp245.Exp.Moy	AX.89438241	6B	704189798	G	C	176	167	9	49.34	10.9991	7.28E-06	5.14	0.3121
19	Sp245.Exp.Med	AX.89438241	6B	704189798	G	C	176	167	9	38.07	7.9310	1.58E-06	5.80	0.3121
19	Sp245.Ind.Moy	AX.89438241	6B	704189798	G	C	174	165	9	0.95	0.2079	4.77E-06	5.32	0.3121
19	Sp245.Exp.Med	AX.89414544	6B	704189826	T	C	176	157	19	27.80	5.6297	7.86E-07	6.10	0.5723
19	Sp245.Exp.Moy	AX.89414544	6B	704189826	T	C	176	157	19	34.69	7.8074	8.84E-06	5.05	0.5723

19	Sp245.Exp.Med	AX.89448288	6B	704302928	A	T	176	160	16	27.43	6.0770	6.39E-06	5.19	0.9266
19	Sp245.Exp.Med	AX.89331766	6B	704303364	G	C	176	161	15	29.07	6.2568	3.39E-06	5.47	0.8336
19	Sp245.Ind.Moy	AX.89725357	6B	704303556	A	G	174	160	14	0.77	0.1693	6.18E-06	5.21	0.5158
19	Sp245.Exp.Med	AX.89772533	6B	704303985	A	G	176	151	25	23.29	5.0044	3.24E-06	5.49	0.0350
19	Sp245.Exp.Med	AX.89499199	6B	704304288	C	T	176	158	18	29.16	5.7656	4.25E-07	6.37	0.8539
19	Sp245.Exp.Moy	AX.89499199	6B	704304288	C	T	176	158	18	36.41	7.9960	5.28E-06	5.28	0.8539
19	Sp245.Exp.Med	AX.89524992	6B	704304308	A	G	176	161	15	29.07	6.2568	3.39E-06	5.47	0.8336
19	Sp245.Exp.Med	AX.89622911	6B	704304351	A	G	176	161	15	29.07	6.2568	3.39E-06	5.47	0.8336
19	Sp245.Ind.Moy	AX.89502932	6B	704305211	T	C	174	160	14	0.77	0.1693	6.18E-06	5.21	0.5158
19	Sp245.Ind.Moy	AX.89410503	6B	704505153	C	T	174	161	13	0.79	0.1751	5.68E-06	5.25	0.2027
19	Sp245.Ind.Moy	AX.89503405	6B	704505213	T	C	173	160	13	0.79	0.1752	5.70E-06	5.24	0.2128
19	Sp245.Exp.Med	AX.89447957	6B	704506160	A	G	176	154	22	26.83	5.2825	3.81E-07	6.42	0.1796
19	Sp245.Exp.Med	AX.89479632	6B	704507056	G	C	176	154	22	25.01	5.2825	2.20E-06	5.66	0.4508
19	Sp245.Ind.Moy	AX.89570443	6B	704507709	T	C	174	160	14	0.77	0.1693	6.18E-06	5.21	0.5158
19	Sp245.Exp.Med	AX.89698906	6B	704508272	C	T	176	146	30	21.45	4.6459	3.91E-06	5.41	0.1764
19	Sp245.Ind.Moy	AX.89428743	6B	704551936	C	T	174	160	14	0.77	0.1693	6.18E-06	5.21	0.5158
19	Sp245.Ind.Moy	AX.89683104	6B	704652484	G	A	174	160	14	0.77	0.1693	6.18E-06	5.21	0.5158
19	Sp245.Ind.Moy	AX.89573171	6B	704704787	A	T	174	161	13	0.83	0.1751	2.44E-06	5.61	0.5146
19	Sp245.Ind.Moy	AX.89358001	6B	704751665	G	T	174	160	14	0.77	0.1693	6.18E-06	5.21	0.5158
19	Sp245.Exp.Med	AX.89426825	6B	704788171	C	T	176	163	13	31.94	6.6795	1.74E-06	5.76	0.6120
19	Sp245.Ind.Moy	AX.89426825	6B	704788171	C	T	174	161	13	0.84	0.1751	1.70E-06	5.77	0.6120
19	Sp245.Ind.Moy	AX.89474943	6B	704790406	G	A	174	160	14	0.77	0.1693	6.18E-06	5.21	0.5158
19	Sp245.Ind.Moy	AX.89590209	6B	704860630	A	T	174	159	15	0.78	0.1641	2.08E-06	5.68	0.4926
19	Sp245.Ind.Med	AX.89590209	6B	704860630	A	T	174	159	15	0.55	0.1232	8.99E-06	5.05	0.4926
19	Sp245.Ind.Moy	AX.89667199	6B	704861385	C	T	174	159	15	0.79	0.1641	1.49E-06	5.83	0.4507
19	Sp245.Ind.Med	AX.89667199	6B	704861385	C	T	174	159	15	0.56	0.1232	5.72E-06	5.24	0.4507
19	Sp245.Ind.Moy	AX.89627592	6B	704882835	C	G	174	160	14	0.77	0.1693	6.18E-06	5.21	0.5158
19	Sp245.Ind.Moy	AX.89587148	6B	704883207	C	G	174	160	14	0.77	0.1693	6.18E-06	5.21	0.5158
19	Sp245.Ind.Moy	AX.89354527	6B	704883683	C	T	174	161	13	0.84	0.1751	1.70E-06	5.77	0.6120
19	Sp245.Exp.Med	AX.89354527	6B	704883683	C	T	176	163	13	31.94	6.6795	1.74E-06	5.76	0.6120
19	Sp245.Ind.Moy	AX.89379712	6B	704884899	C	A	174	160	14	0.77	0.1693	6.18E-06	5.21	0.5158

19	Sp245.Exp.Med	AX.89712251	6B	704885060	T	C	176	163	13	30.66	6.6795	4.42E-06	5.35	0.5146
19	Sp245.Ind.Moy	AX.89708135	6B	704886333	G	A	174	160	14	0.77	0.1693	6.18E-06	5.21	0.5158
19	Sp245.Ind.Moy	AX.89550611	6B	704886431	T	C	174	160	14	0.77	0.1693	6.18E-06	5.21	0.5158
19	Sp245.Ind.Moy	AX.89411948	6B	704886786	A	G	174	160	14	0.77	0.1693	6.18E-06	5.21	0.5158
19	Sp245.Ind.Moy	AX.89321148	6B	704888976	T	A	174	160	14	0.77	0.1693	6.18E-06	5.21	0.5158
19	Sp245.Ind.Moy	AX.89421975	6B	704945976	T	C	174	160	14	0.77	0.1693	6.18E-06	5.21	0.5158
19	Sp245.Ind.Moy	AX.89735767	6B	704949137	C	T	174	160	14	0.77	0.1693	6.18E-06	5.21	0.5158
19	Sp245.Ind.Moy	AX.89496681	6B	704949468	G	A	174	160	14	0.77	0.1693	6.18E-06	5.21	0.5158
19	Sp245.Ind.Moy	AX.89509555	6B	704949487	T	A	174	160	14	0.77	0.1693	6.18E-06	5.21	0.5158
19	Sp245.Ind.Moy	AX.89526184	6B	704964940	C	T	174	160	14	0.77	0.1693	6.18E-06	5.21	0.5158
19	Sp245.Ind.Moy	AX.89405827	6B	704965201	G	A	174	159	15	0.80	0.1641	1.20E-06	5.92	0.4926
19	Sp245.Ind.Med	AX.89405827	6B	704965201	G	A	174	159	15	0.57	0.1232	3.50E-06	5.46	0.4926
19	Sp245.Ind.Moy	AX.89733441	6B	704976010	G	A	174	160	14	0.77	0.1693	6.18E-06	5.21	0.5158
19	Sp245.Exp.Med	AX.89761693	6B	705139561	T	C	176	163	13	30.66	6.6795	4.42E-06	5.35	0.5146
19	Sp245.Ind.Moy	AX.89357867	6B	705282230	A	G	174	160	14	0.77	0.1693	6.18E-06	5.21	0.5158
19	Sp245.Ind.Moy	AX.89438186	6B	705312685	G	A	174	156	18	0.67	0.1512	9.44E-06	5.03	0.6588
19	Sp245.Ind.Moy	AX.89314150	6B	705314997	G	C	174	155	19	0.67	0.1476	4.88E-06	5.31	0.8704
20	Sp245.Ind.Moy	AX.89361492	6B	159410401	G	A	174	163	11	0.86	0.1892	5.71E-06	5.24	0.9574
21	Sp245.Exp.Med	AX.89500210	7A	30092884	G	A	175	147	28	21.42	4.7790	7.41E-06	5.13	**0.0019**
22	Sp245.Exp.Med	AX.89644216	7D	93078495	A	G	176	160	16	27.24	6.0770	7.37E-06	5.13	0.3385
22	Sp245.Exp.Med	AX.89622716	7D	93094670	T	A	176	160	16	27.87	6.0770	4.51E-06	5.35	0.5848
22	Sp245.Exp.Med	AX.89536477	7D	93603574	A	T	176	161	15	30.72	6.2568	9.13E-07	6.04	0.5848
22	Sp245.Exp.Med	AX.89512638	7D	93605114	T	C	176	160	16	27.24	6.0770	7.37E-06	5.13	0.3385

† Using screening data in Valente et al. (2019) for *Pseudomonas kilonensis* F113, only one genetic marker (related to colonization rate) was identified (Trait, F113.Col.Moy; Marker, AX.89765457; Chromosome, 1A; Position, 14114969; A, A; B, T; NoMeasured, 178; NbA, 154; NbB, 24; effB, 10.09; SE_effB, 2.26; p_Marker, 8.14E-06; $-\log_{10}(P)$, 5.09).
